# Microneedle-Assisted Cell Delivery and Therapy

**DOI:** 10.34133/research.1382

**Published:** 2026-08-04

**Authors:** Ruochen Qiao, Dasheng Li, Long Wang, Chenxuan Zhao, Bin Kong, Jinhui Wu

**Affiliations:** ^1^ State Key Laboratory of Pharmaceutical Biotechnology, Medical School of Nanjing University, Nanjing University, Nanjing 210093, China.; ^2^ Research Center for High Altitude Medicine, Xining 810000, China.; ^3^ Department of Thoracic Surgery, Nanjing Drum Tower Hospital, The Affiliated Hospital of Nanjing University Medical School, Nanjing 210031, China.; ^4^ School of Biomedical Engineering, Shenzhen University Medical School, Shenzhen University, Shenzhen 518060, China.

## Abstract

Cell-based therapeutics hold immense promise for clinical intervention, yet their practical translation is severely bottlenecked by suboptimal delivery kinetics, safety risks, and low patient adherence. This review provides a comprehensive overview of recent innovations in microneedle (MN)-based cell delivery systems and their applications across multiple organs. We initiate the discussion by evaluating progress in regenerative medicine, specifically targeting stem and immune-derived lineages. The article further classifies MN systems for cell therapy delivery according to material composition, fabrication techniques, and functional characteristics, with an in-depth discussion of innovative designs, highlighting their unique advantages. Additionally, we categorize and analyze recent studies on MN-based cell therapy for skin diseases, endometrial repair, cardiac regeneration, and oral disorders. Beyond summarizing current achievements, we critically examine the translational barriers that limit clinical adoption, including manufacturing scalability, storage stability, and regulatory considerations. This review provides a systematic and organ-specific overview that may help guide the design of improved and more clinically translatable cell delivery platforms.

## Introduction

Cell-based therapy, also termed as cell therapy, involves administering viable cells to achieve therapeutic effects across a range of diseases [1]. This approach offers multiple advantages over traditional surgical interventions and pharmaceutical treatments. Owing to the controllable release of therapeutic cells and their sustained contact with human tissues, cell therapy ensures long-term efficacy [2]. The administered cells generate secretions such as cytokines, which contribute to host defense, target damaged cells for restoration, and promote tissue repair through anti-apoptotic, anti-inflammatory, and pro-angiogenic functions [3]. Furthermore, cell therapy addresses several obstacles of conventional treatment, including immunogenicity, oncogenic transformation, and high cost [4]. Despite considerable technological and biological advances, as well as demonstrated ease, safety, and efficacy of administration, the clinical translation and commercialization of cell therapy remain challenging [5]. Specifically, inconvenience of handling, low patient compliance, delivery efficiency, and inadequate biosafety collectively restrict developmental progress [6]. In response, microneedles (MNs) have gained attention as an effective delivery approach, characterized by painless administration, reduced tissue disruption, and user-friendly operation [7].

MNs function as microscopic administration platforms, typically utilizing arrays with a longitudinal reach of 25 to 1,000 μm [8]. Their minute scale facilitates the disruption of outer skin barriers, particularly the stratum corneum, without contacting blood vessels or nerve endings to ensure a virtually painless application. Hence, MNs overcome the major problems associated with the traditional transdermal administration methods, such as topical creams, hypodermic needles, and transdermal patches [9]. Additional advantages of MN-based systems include targeted and rapid delivery, improved patient compliance, and tunable release kinetics achieved through material selection and structural design [10]. Notably, MN-mediated delivery has facilitated the clinical translation of cell therapies, demonstrating marked therapeutic effects across various diseases [11].

**Fig. 1.**
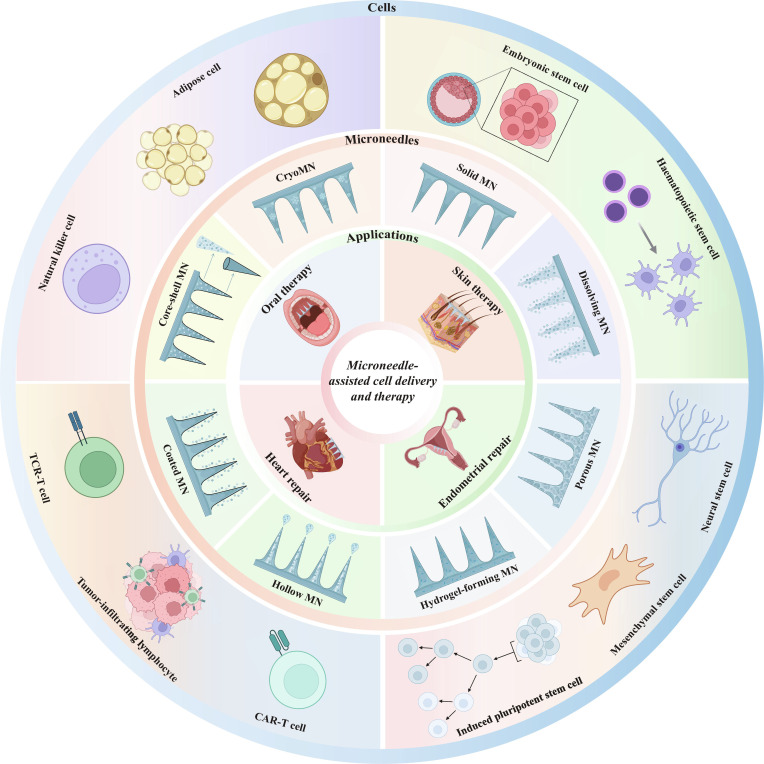
Loaded cell types, MN types, and biomedical applications of MN-mediated cell therapy. Illustration created in BioRender.

Herein, this article reviews MN-assisted cell delivery and their therapeutic use across various organs. We first outline recent progress in cell therapy, specifically concentrating on stem cells (SCs) and immune cells. We then summarize developments in MN technology based on fabrication materials, methods, and specific characteristics, providing a detailed discussion of innovative types, such as porous MNs, hydrogel-forming MNs, and cryomicroneedles (cryoMNs), along with their respective advantages. Furthermore, we categorize the latest research on MN-mediated cell therapy for skin diseases, endometrial repair, heart repair, and oral diseases. Unlike previous reviews that primarily summarize existing literature, this article provides a critical assessment of current achievements, identifies key translational obstacles, and discusses strategies to address manufacturing, regulatory, and clinical implementation challenges. In closing, we analyze the principal barriers to clinical implementation and propose forward-looking solutions to guide future investigation (Fig. 1).

## Recent Progress in Cell Therapy

### SC therapy

**Fig. 2.**
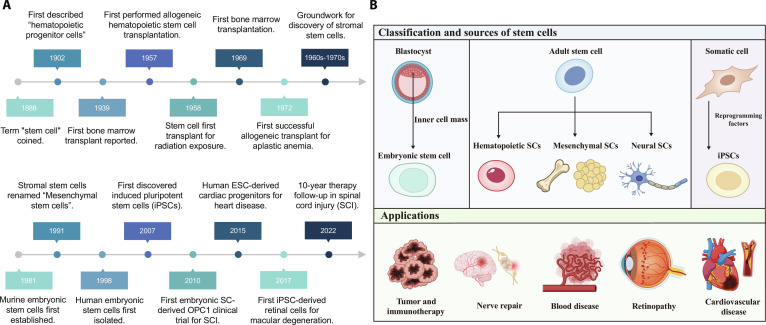
SC-based therapies: historical milestones, cellular sources, and promising application directions. (A) Timeline of the essential progress made in scientific inquiry and practical implementation regarding SC-mediated therapeutic strategies. Reproduced with permission from Ref. [17]. Copyright The Authors 2022. (B) The diagram depicts the hierarchical classification of SC subsets based on their ontogeny and lineage potency, highlighting diverse therapeutic modalities and emerging frontiers in regenerative medicine. Created in BioRender.com.

SCs are undifferentiated, self-renewing biological units whose intrinsic plasticity forms the cornerstone of modern regenerative medicine [12]. They are widely distributed across diverse anatomical niches, including the bone marrow, adipose tissue, and umbilical cord [13]. These precursors offer transformative approaches for managing complex pathologies like autoimmune reactions, cutaneous injuries, and chronic organ dysfunction. Current clinical investigation heavily focuses on leveraging these progenitors for targeted immune regulation, cellular reconstitution, and intrinsic neurovascular remodeling [14,15]. Driven by next-generation technologies such as viral vector-mediated transduction, contemporary research enables sophisticated genetic and functional modifications to engineer SCs into refined, personalized therapeutic platforms [16]. Ultimately, ongoing research continues to elucidate the precise functional attributes and subclassifications of these lineages, marking pivotal strides toward long-term organ recovery and robust clinical translation across the wider regenerative medicine landscape (Fig. 2) [17].

#### Embryonic SC therapy

Embryonic SCs (ESCs) possess pluripotent differentiation capacity and have shown clinical proof-of-concept in ophthalmology and neurology, where ESC-derived retinal pigment epithelium and dopaminergic progenitors achieved graft survival and functional integration in patients with macular degeneration and Parkinson’s disease, respectively [18,19]. However, this success is not generalizable to complex solid organs, and conventional injection causes massive cell loss and poor distribution beyond inherent teratoma and immune risks [20]. Thus, ESC therapy remains disease-specific and urgently requires precise, cytoprotective delivery platforms [21].

#### Adult SC therapies

Adult SCs (ASCs) are multipotent progenitors resident within postnatal tissues, offering enhanced biosafety, reduced oncogenic risk, and minimal ethical impediments compared to ESCs [22]. In the context of targeted regenerative medicine, 4 primary lineages of ASCs are systematically utilized based on their distinct anatomical origins and therapeutic properties [23].

**Fig. 3.**
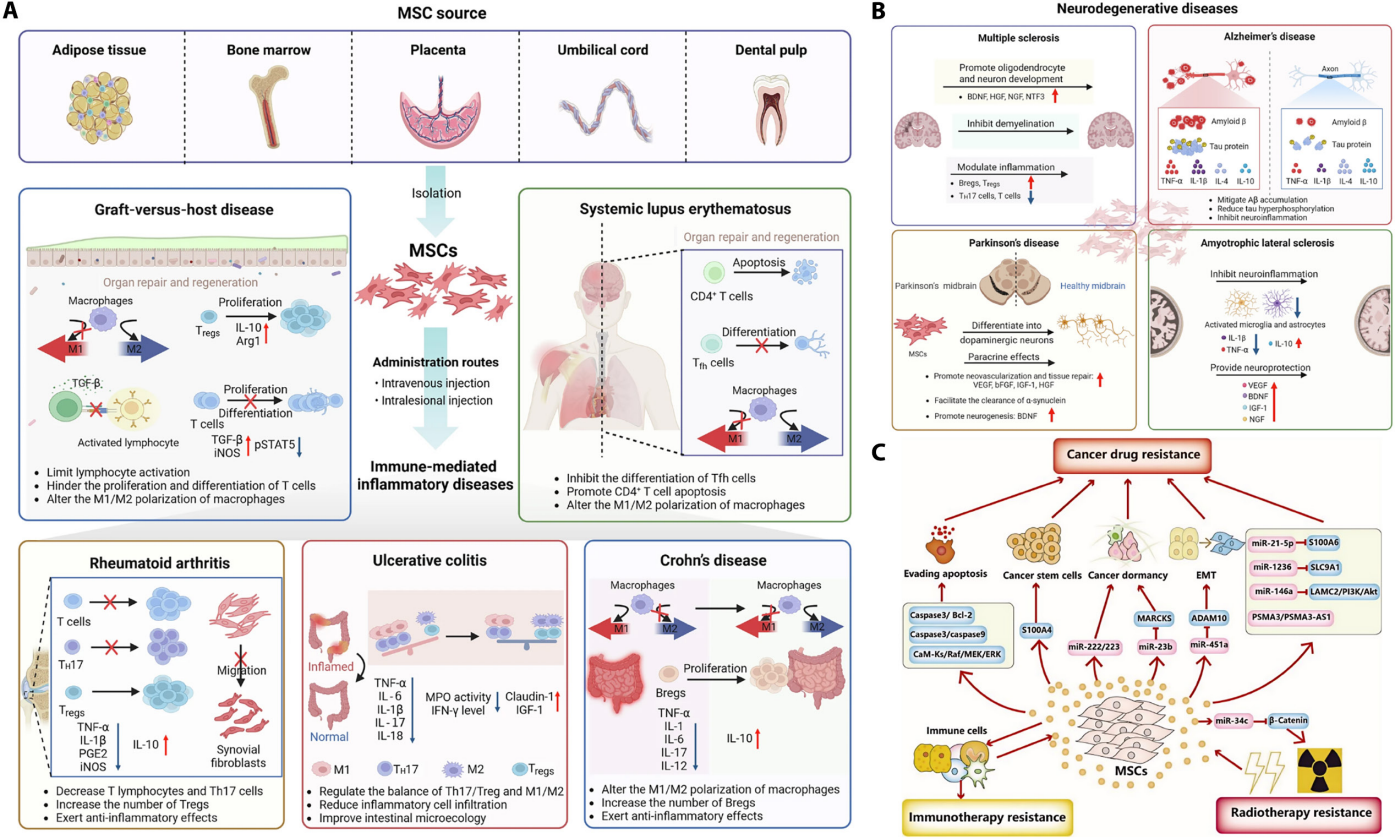
Therapeutic applications of MSCs from various origins. MSCs harvested from fatty deposits, skeletal marrow, dental structures, and birth-associated tissues demonstrate broad clinical efficacy. (A) Regulatory pathways of these units in immune-related inflammatory conditions. This schematic depicts the mitigation of chronic autoimmune and gastrointestinal ailments, such as lupus, rheumatoid joint inflammation, and inflammatory bowel diseases. (B) Neuroprotective mechanisms of MSC therapy in managing neurodegenerative disorders, including various Parkinsonian, Alzheimer-type, and paralytic disorders. Reproduced with permission from Ref. [28]. Copyright The Authors 2025. (C) Paradoxical role of MSC-derived exosomes in malignancy treatment. They drive treatment failure by inhibiting apoptosis, modulating cancer SCs, and regulating dormancy, impacting response to drug, immuno-, and radiotherapy. Reproduced with permission from Ref. [29]. Copyright The Authors 2022.

Firstly, hematopoietic SCs (HSCs), typically harvested from bone marrow or umbilical cord blood, represent the most clinically mature class of ASCs [24]. They possess a unique homing ability and the capacity to reconstitute the entire blood and immune systems, making them the gold standard for treating hematological malignancies [25]. However, achieving efficient systemic homing without high-dose pre-conditioning regimens remains a major delivery challenge [26]. Secondly, mesenchymal SCs (MSCs) are self-renewing, multipotent progenitors whose therapeutic utility spans inflammatory, neurological, and oncological applications primarily via powerful, source-dependent paracrine mechanisms (Fig. 3) [27,28]. Despite this broad clinical potential, MSC therapies currently exhibit variable efficacy due to tissue-specific biological variations, harvesting limitations, and complex microenvironmental interactions [29]. Consequently, the successful clinical translation of MSC-based interventions remains highly context-dependent, necessitating advanced strategies to optimize cell retention, targeted delivery, and sustained paracrine activity at the diseased site [30].

Thirdly, neural SCs (NSCs) hold specialized promise for treating ischemic stroke and neurodegenerative disorders by migrating to injured regions to reconstruct neuronal circuits and modulate the local pathological microenvironment. However, their translation into mainstream clinical use remains bottlenecked by highly variable efficacy that is heavily influenced by disease pathology, delivery routes, and the rigorous requirements for sustained graft viability and effective synaptic incorporation. Consequently, advancing NSC therapies necessitates targeted strategies to optimize regional cell retention, survival, and long-term functional integration [31,32]. Lastly, adipose-derived SCs (ADSCs), isolated efficiently via minimally invasive liposuction, provide a plentiful and highly proliferative multipotent cargo that bypasses ethical constraints [33]. Rather than merely serving as energy storage, these cells accelerate tissue repair and angiogenesis through the robust paracrine secretion of vital factors like vascular endothelial growth factor (VEGF), insulin-like growth factor 1 (IGF-1), and extracellular vesicles [34,35]. Consequently, ADSCs have seen extensive clinical translation in wound healing, reconstructive surgery, and craniofacial reconstruction, where they are frequently combined with osteoinductive cytokines and biomaterial scaffolds like tricalcium phosphate and hydrogels to further enhance tissue regeneration [36].

#### Induced pluripotent SC therapy

Generated by reprogramming somatic cells, induced pluripotent SCs (iPSCs) offer unlimited growth and broad differentiation without embryonic ethical concerns, facilitating patient-specific therapies with improved immune acceptance [14]. Although promising for cardiovascular, neurological, and retinal disorders, iPSC clinical translation is severely bottlenecked by tumorigenicity, genetic instability, and variable differentiation efficiency [37,38]. Consequently, despite advancements like CRISPR-Cas9, achieving broad clinical viability relies on ongoing improvements in manufacturing consistency, cellular safety, and delivery strategies that maximize post-transplantation survival and local engraftment [39,40].

Emerging clinical data indicate that SC outcomes depend on matching the cell lineage, disease pathology, target tissue biology, and administration route. Among current modalities, HSC interventions are the most clinically validated; for example, exagamglogene autotemcel successfully eliminated crises in severe sickle cell disease. However, HSC success remains largely restricted to hematological disorders and demands complex ex vivo processing and clinical infrastructure [41]. Conversely, MSC therapies exhibit favorable safety but inconsistent efficacy in complex tissue injuries. In the TREASURE phase 2/3 trial for acute ischemic stroke, intravenous allogeneic therapy failed to markedly improve short-term functional outcomes over placebo, demonstrating that broad paracrine or immunomodulatory activity alone cannot guarantee clinical reproducibility amidst heterogeneous pathology and limited cell retention [42]. NSC and iPSC-derived lineages face even steeper translational hurdles regarding functional integration and delivery precision. The PISCES III trial for post-stroke disability underscored the friction of invasive stereotactic intracerebral NSC administration [43]. Similarly, while iPSC-derived dopaminergic progenitors showed safety and graft survival in Parkinson’s patients, the approach remains strictly disease-specific, requiring precise lineage differentiation and accurate spatial deployment [44]. Collectively, these paradigms show that SCs are not universally applicable. While HSCs excel in accessible hematological systems and MSCs offer safe but variable immunomodulation, NSC and iPSC therapies remain bottlenecked by spatial constraints and long-term functional integration. Consequently, refining localized cell delivery, survival, and tissue incorporation is paramount to achieving reproducible clinical viability.

### Immune cell therapy

**Fig. 4.**
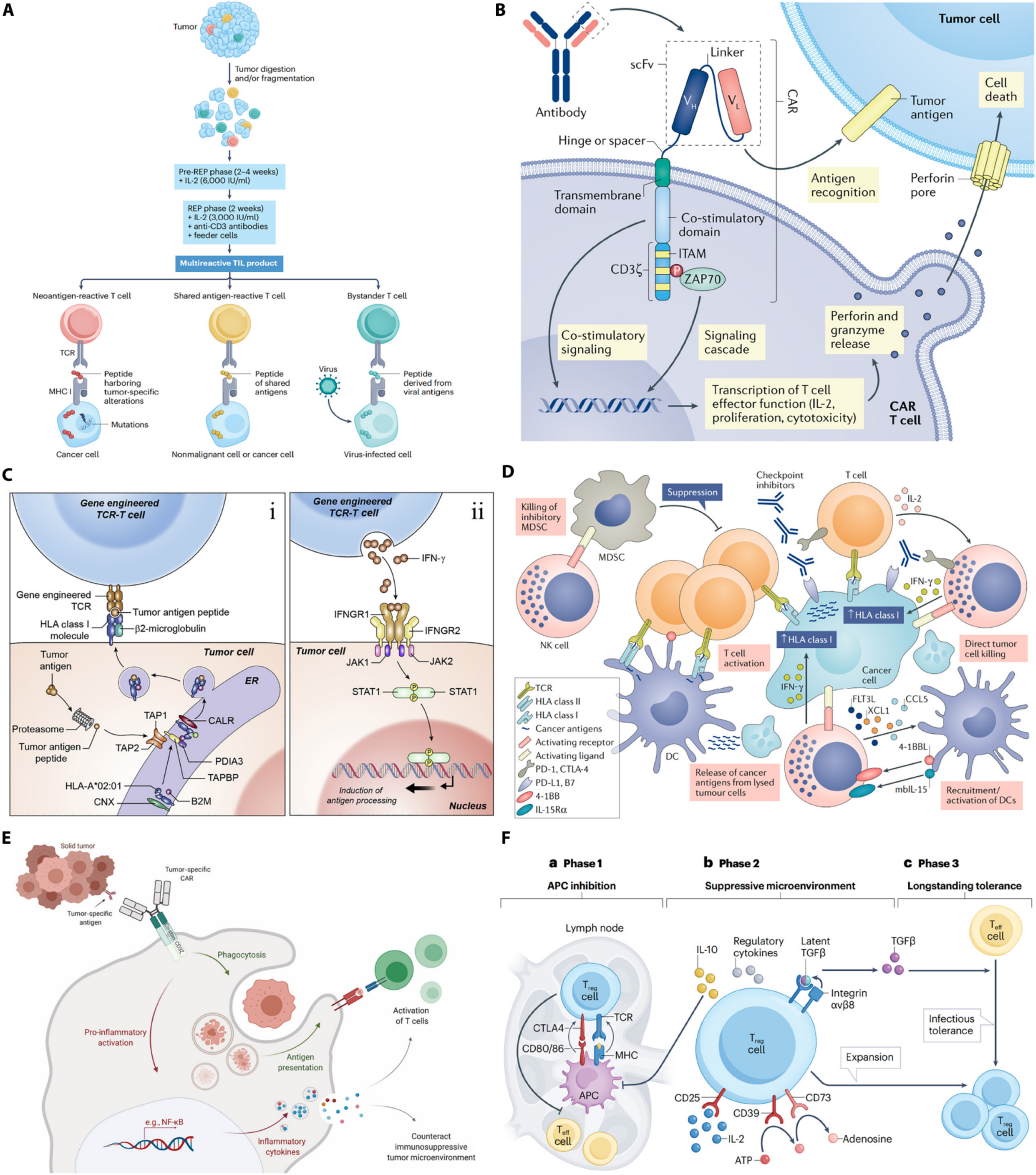
Cancer immunotherapy utilizing immune cell-based approaches. (A) Schematic depicting the manufacturing process of a polyreactive TIL cell product for therapeutic use, derived from metastatic lesion tissue. Reproduced with permission from Ref. [48]. Copyright Springer Nature Limited 2024. (B) A diagram demonstrates how CAR-T cells operate, depicting the selective engagement of neoplastic antigens via specialized receptor domains to facilitate the eradication of malignant cells. Reproduced with permission from Ref. [123]. Copyright Springer Nature Limited 2021. (C) Essential mechanisms of TCR-mediated interventions, highlighting the recognition and elimination of tumor cells, specifically (i) the interaction with peptide–MHC assemblies presented on the target membrane and (ii) the subsequent activation of cytotoxic responses by the modified T lymphocytes. Reproduced with permission from Ref. [163]. Copyright 2022 Elsevier Inc. (D) Diagram illustrating NK cell-based immunotherapy, covering both their intrinsic capacity to lyse tumor cells directly and their contribution to amplifying the antitumor activity of other immune populations. Reproduced with permission from Ref. [164]. Copyright Springer Nature Limited 2020. (E) Activation and anti-cancer cascade of macrophage therapy. Reproduced with permission from Ref. [74]. Copyright Authors 2021. (F) Overview of the 3 sequential phases in T_reg_ cell therapy for mediated immunosuppression. Reproduced with permission from Ref. [62]. Copyright Springer Nature Limited 2024.

Immunotherapeutic modalities are broadly categorized into 6 primary groups determined by their functional mechanisms, encompassing tumor-infiltrating lymphocytes (TILs), chimeric antigen receptor T (CAR-T) cells, T cell receptor-engineered T (TCR-T) cells, and natural killer (NK) cells, macrophages, and regulatory T (T_reg_) cells [45]. Often referred to as “living therapies” to distinguish them from conventional medicines, these strategies have yielded profound therapeutic successes in addressing diverse malignancies and specific viral pathogens [46], as illustrated in Fig. 4. Synergistic progress in genomic modification and synthetic biology is continuously broadening the scope and optimizing the potency of these cellular interventions [47]. This section evaluates the modern landscape, existing hurdles, and prospective outlook for these transformative immune-based protocols.

#### T cell-based therapies

T cell-based therapies constitute one of the most extensively developed branches of adoptive cellular therapy and can be broadly divided into TIL therapy, CAR-T therapy, TCR-T therapy, and T_reg_ therapy. Although these strategies all rely on the therapeutic use of T cells, they differ substantially in antigen-recognition mode, engineering strategy, clinical indication, and therapeutic intent.

TIL therapy uses autologous lymphocytes isolated from tumor lesions, followed by ex vivo expansion and reinfusion into the patient [48]. Unlike CAR-T and TCR-T therapies, which usually rely on a defined engineered receptor, TIL products are naturally polyclonal and can recognize multiple tumor-associated or tumor-specific antigens, including neoantigens, cancer–testis antigens, differentiation antigens, and virus-associated antigens [49,50]. This broad antigen-recognition capacity may help address tumor heterogeneity and antigen escape in solid tumors. Clinically, metastatic melanoma remains the most validated indication. Lifileucel achieved durable responses in advanced melanoma and received the Food and Drug Administration (FDA) accelerated approval in 2024 for unresectable or metastatic melanoma after PD-1 inhibitor progression, becoming the first approved TIL product and the first approved T cell therapy for a solid tumor indication [51,52]. TIL therapy is also being explored in other solid tumors, including lung, cervical, ovarian, gastrointestinal, head and neck, breast, colorectal, renal, and sarcoma indications.

CAR-T therapy redirects T cells through synthetic chimeric antigen receptors that recognize tumor surface antigens in a major histocompatibility complex (MHC)-independent manner [53,54]. Most approved products use second-generation CAR designs containing CD3ζ and either CD28 or 4-1BB costimulatory domains, which respectively favor rapid activation or long-term persistence. CAR-T therapy has achieved the greatest clinical success in relapsed or refractory hematological malignancies. As of 2026, approved products include CD19-directed CAR-T cells, such as tisagenlecleucel, axicabtagene ciloleucel, brexucabtagene autoleucel, obecabtagene autoleucel, and lisocabtagene maraleucel, as well as B cell maturation antigen (BCMA)-directed products, including idecabtagene vicleucel and ciltacabtagene autoleucel. Their approved indications now cover B cell acute lymphoblastic leukemia, diffuse large B cell lymphoma and related subtypes, mantle cell lymphoma, follicular lymphoma, chronic lymphocytic leukemia/small lymphocytic lymphoma, and multiple myeloma. More recently, CAR-T therapy has begun to extend into solid tumors; satricabtagene autoleucel, a CLDN18.2-targeted CAR-T product, was approved in China for CLDN18.2-positive advanced gastric or gastroesophageal junction adenocarcinoma, representing an important milestone for CAR-T therapy beyond hematological malignancies [55–57]. Current development is focused on overcoming antigen escape, limited solid tumor infiltration, suppressive tumor microenvironments (TMEs), T cell exhaustion, and individualized manufacturing barriers through dual-target CARs, armored CAR-T cells, gene-edited allogeneic products, and in situ CAR-T generation strategies.

TCR-T therapy introduces genes encoding tumor-specific TCRs into patient T cells, enabling recognition of intracellular antigen-derived peptides presented by MHC molecules. This feature expands the targetable antigen repertoire beyond cell-surface proteins to include cancer–testis antigens, lineage-associated antigens, viral antigens, and selected neoantigens [58]. However, TCR-T efficacy depends on specific peptide–human leukocyte antigen (HLA) recognition, thereby restricting eligibility to patients with compatible HLA alleles [59]. Compared with CAR-T cells, TCR-T cells can respond to relatively low densities of peptide–MHC complexes, reflecting the sensitivity of physiological TCR signaling [60]. Clinically, TCR-T therapy has shown particular promise in solid tumors. Afamitresgene autoleucel, an HLA-A*02-restricted melanoma-associated antigen 4 (MAGE-A4)-specific TCR-T therapy, received FDA accelerated approval in 2024 for unresectable or metastatic synovial sarcoma after prior chemotherapy, marking an important milestone for engineered TCR-based therapy in solid tumors [61].

In contrast to TIL, CAR-T, and TCR-T therapies, which are designed mainly to enhance antitumor immunity, T_reg_ therapy aims to restore immune tolerance. T_reg_ cells are immunoregulatory T cells characterized by high CD25 expression, low CD127 expression, and FOXP3-dependent lineage stability. They suppress immune activation through CTLA-4-mediated reduction of CD80/CD86 costimulation, secretion of interleukin-10 (IL-10), transforming growth factor-β (TGF-β), and IL-35, CD39/CD73-mediated adenosine production, and CD25-dependent IL-2 consumption, thereby limiting effector T cell expansion and persistent inflammation [62]. Accordingly, T_reg_ therapy is being developed primarily for autoimmune diseases, organ transplantation, graft-versus-host disease, and other inflammatory disorders. Early clinical studies in type 1 diabetes, kidney transplantation, and HSC transplantation have shown that ex vivo-expanded polyclonal T_reg_ cells or induced T_reg_ cells are generally feasible and well tolerated, although durable efficacy remains limited by cell persistence, tissue homing, product stability, and insufficient antigen specificity [63–65].

Overall, TILs exploit naturally occurring polyclonal tumor reactivity, CAR-T cells provide MHC-independent recognition of surface antigens, TCR-T cells extend targeting to intracellular peptide–MHC antigens, and T_reg_ cells represent a tolerance-restoring platform for immune-mediated diseases. Together, these complementary approaches have expanded the therapeutic scope of T cell-based interventions from hematologic malignancies to solid tumors, autoimmunity, transplantation, and inflammatory disorders.

#### NK cell therapy

NK cells are cytotoxic innate lymphocytes that distinguish abnormal cells from healthy tissues by integrating signals from inhibitory and activating receptors. Unlike T cells, NK cell activity is governed by balancing MHC-I recognition and stress-ligand sensing. Beyond direct cytotoxicity via perforin, granzymes, and CD16-mediated antibody-dependent cellular cytotoxicity, NK cells secrete cytokines like interferon-γ (IFN-γ) and tumor necrosis factor-α (TNF-α) to orchestrate TME remodeling and T cell recruitment [2]. NK-based immunotherapies exploit this biology through diverse modalities, including unmodified, cytokine-primed memory-like, and engineered CAR-NK cells [66]. CAR-NK platforms often incorporate enhancements such as IL-15 modules, suicide switches, and logic-gated circuits to improve persistence, trafficking, and resilience against immunosuppressive tumor environments [67]. Notably, iPSC-derived NK cells are particularly promising for off-the-shelf development, offering clonal uniformity, scalable manufacturing, and compatibility with multiplex gene editing [68]. Clinically, NK cell therapies are being investigated across a broad spectrum of hematologic malignancies and solid tumors, such as acute myeloid leukemia, multiple myeloma, glioblastoma, pancreatic, and breast cancer [69]. While these therapies have not yet achieved the regulatory maturity of CAR-T platforms, their favorable safety profiles, allogeneic compatibility, and intrinsic antitumor potential position them as a robust, standardized complement to existing cancer immunotherapies [70].

#### Macrophage-based cell therapy

Macrophages are tissue-resident innate immune cells capable of phagocytosis, antigen presentation, inflammatory regulation, and tissue repair. These properties enable macrophage-based therapies to remodel diseased tissue microenvironments. While ex vivo-polarized macrophages are being evaluated in liver cirrhosis and chronic anal fissures, chimeric antigen receptor macrophages (CAR-M) have emerged as a prominent platform for solid tumors [71]. Unlike CAR-T or CAR-NK therapies, CAR-M combine antigen recognition with macrophage-specific effector functions. Their therapeutic activity extends beyond direct tumor clearance to include repolarizing the TME from an immunosuppressive to a pro-inflammatory state, thereby activating downstream dendritic cell (DC)- and T cell-mediated immunity [72]. This dual capability makes CAR-M attractive for solid malignancies where immune exclusion and stromal barriers limit conventional T cell approaches. Clinically, CT-0508, a human epidermal growth factor receptor 2 (HER2)-targeted CAR-M, has entered phase I trials for HER2-overexpressing solid tumors [73]. Early results demonstrated favorable tolerability without dose-limiting toxicities, severe cytokine release syndrome, or neurotoxicity. Multi-omic analyses confirmed successful tumor localization, microenvironment remodeling, and local CD8^+^ T cell expansion, providing clinical validation for the safety, feasibility, and biological activity of CAR-M therapy in solid tumors [74].

### Delivery bottlenecks in current cell therapy

Despite the documented clinical success of specialized indications like CAR-T cell therapy and HSC transplantation, the broad efficacy of most cell-based interventions remains heavily constrained by delivery-associated barriers rather than cellular biology alone [75]. As fragile therapeutic agents, living cells undergo severe shear and tensile stresses during needle injection, compromising membrane integrity and compromising immediate viability. Upon transplantation, these cells encounter an inhospitable microenvironment characterized by hypoxia, nutrient deprivation, oxidative stress, acute inflammation, and a lack of immediate vascularization, which collectively precipitate massive early cell mortality [76–78]. Furthermore, ex vivo expansion and subsequent administration as single-cell suspensions disrupt native cell–matrix and cell–cell interactions, rendering cells highly susceptible to anoikis and functional impairment. Even viable cells suffer from severe local attrition due to leakage along the injection tract, passive interstitial diffusion, lymphatic drainage, and poor adhesion to the host extracellular matrix (ECM), culminating in exceptionally low rates of long-term engraftment and functional persistence [75,79].

These delivery bottlenecks carry profound clinical and manufacturing consequences. Poor retention and rapid clearance necessitate repeated or high-dose administrations, escalating patient discomfort, procedural risks, and financial costs, while uncontrolled spatial migration compromises therapeutic precision and elevates off-target safety risks. From a chemistry, manufacturing, and controls (CMC) perspective, the requirement for inflated cellular doses severely compounds the complexity of Good Manufacturing Practice (GMP)-compliant workflows. Because living cellular products demand rigorous standardization regarding identity, purity, potency, and batch-to-batch consistency, minimizing biological cell loss through enhanced delivery efficiency is a prerequisite for scalable, cost-effective clinical manufacturing [80,81]. Consequently, advancing cell therapy requires a paradigm shift toward integrated delivery platforms capable of protecting cellular cargo during administration, mitigating microenvironmental stress, and ensuring localized tissue incorporation. Among emerging technologies, MN-based arrays offer a highly sophisticated solution by enabling minimally invasive, spatially controlled, and cytoprotective cell deployment. By attenuating insertion-induced mechanical trauma, preventing immediate anatomical leakage, and establishing a localized reservoir within target tissues, MN platforms resolve the prominent shortcomings of conventional injections, representing a pivotal mechanism for improving the consistency, safety, and translational viability of regenerative therapeutics [11,82].

## MN Platforms for Viable Cell Delivery

MN devices are patch-based systems comprising arrays of micrometer-scale needles that integrate features of both transdermal patches and hypodermic needles. By forming microscale pathways across the cutaneous barrier, MNs allow therapeutic payloads to traverse the epidermis and reach the dermis, from which they enter the bloodstream with negligible resistance [83]. As a minimally invasive, precise, and mechanically protective delivery platform, MNs substantially improve the engraftment and survival of delivered cells, overcoming the tissue-targeting deficit inherent to systemic delivery. This localized approach thus amplifies therapeutic potency at the disease site and supports the clinical translation of cell-based interventions. Unlike conventional drug delivery, MN-mediated cell therapy must concurrently address delivery efficiency, cellular viability, and locally controlled release [84]. Living cells exhibit pronounced sensitivity to the local microenvironment provided by MN materials, including factors such as adequate oxygen and moisture supply and appropriate culture temperature. Consequently, MN design for cell delivery should be tailored to the specific disease under treatment, ensuring cell survival and controlled release while synergistically augmenting the therapeutic functions of the delivered cells [11]. To achieve efficient cell delivery and overcome the practical challenges of cell therapy, MN systems must satisfy several critical requirements. Mechanical robustness is essential for tissue penetration, while the platform must also promote cell localization and proliferation at target sites and enable on-demand controlled release tailored to disease-specific needs [82]. Equally important are the preservation of post-administration cell survival and activity, and the retention of cell-specific functions, including cytokine secretion, immune cell recruitment, and T cell-mediated tumor immunity.

**Fig. 5.**
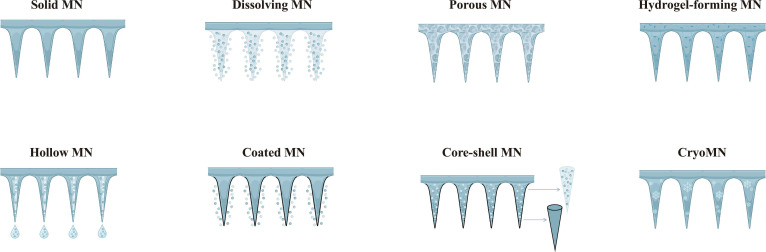
Comparative design characteristics and functional mechanisms of various MN types for cell delivery. Created in BioRender.com.

The rational design of MN platforms for cell delivery hinges on 3 interconnected principles: material selection, structural engineering, and biological integration. Material selection prioritizes biocompatibility, biodegradability, and the mechanical integrity needed for effective skin insertion. Structural engineering tailors the internal architecture by adopting porous, core–shell, or cryogenic configurations, which effectively shield encapsulated cells from shear forces, osmotic insults, and desiccation damage [11]. Biological integration ensures that the delivered cells establish functional interactions with the host microenvironment, enabling proliferation, migration, and therapeutic action. Guided by these principles, the material and structural design of MN platforms must be optimized according to cell type, target tissue, and disease context, with key objectives encompassing cell encapsulation, maintenance of viability, establishment of a cell-supportive microenvironment, and enhancement of therapeutic efficacy through functional integration [1]. In response to diverse cell delivery demands, the MN landscape has expanded considerably in recent years. Eight principal MN subtypes have been established, including solid MNs, dissolving MNs, porous MNs, hydrogel-forming microneedles (HFMs), hollow MNs, coated MNs, core–shell MNs, and cryoMNs, as presented in Fig. 5. These subtypes are characterized by pronounced differences in material composition, fabrication routes, and functional modalities, and the ensuing sections provide a critical appraisal of their respective strengths, vulnerabilities, and performance attributes pertinent to cell delivery.

### Solid MNs

Solid MNs are generally made of silicon, synthetic polymers, or metallic materials such as titanium and stainless steel. These materials confer substantial mechanical resilience, enabling the devices to traverse compact tissue barriers while preserving their geometric integrity [85]. However, their inherently dense and aperture-free architecture precludes the internal encapsulation of living cells, thereby restricting their direct application in cell delivery to surface-functionalization strategies or pretreatment roles [86]. For instance, cellular components can be delivered either via surface coating techniques or by applying a cell-loaded patch over the microchannels generated by pre-piercing, which has subsequently given rise to coated and dissolving MN variants [87]. In parallel, surface functionalization of solid MNs with bioactive cues such as chemokines or antibodies has been explored to recruit host cells in situ, although it has yet to demonstrate reliable therapeutic efficacy [88]. Despite these innovations, the intrinsic incapacity of solid MNs to enable sustained, high-dosage, and viable delivery of exogenous cells remains a major obstacle, particularly in the contexts of complex regenerative medicine and adoptive immunotherapies. Moreover, concerns over suboptimal biocompatibility and increased susceptibility to infection, attributable to certain material choices and structural simplicity, have further diminished their translational appeal, increasingly favoring next-generation MN platforms with enhanced functionality and biosafety profiles [89].

### Dissolving MNs

Dissolving MNs function via a “poke-and-release” mechanism, wherein active pharmaceutical ingredients are embedded directly into a biodegradable, water-soluble polymer framework, often fabricated from materials like polyvinylpyrrolidone (PVP) and polyvinyl alcohol (PVA) [90]. Upon skin insertion, the matrix dissolves in situ through absorption of interstitial fluid (ISF), enabling localized cargo deposition while circumventing the generation of sharp infectious waste [8,91]. Although initially optimized for small molecules and macromolecules, dissolving MNs have recently been repurposed to address critical delivery bottlenecks in cellular and cell-derived therapies, including poor cellular retention, systemic off-target effects, and stringent cold-chain dependence [84,92]. A primary advantage lies in their capacity to function as a solid-state stabilizing scaffold for fragile cargoes such as extracellular vesicles and exosomes. For instance, encapsulating SC-derived vesicles within a hyaluronic acid (HA)-based dissolving MNs matrix has been shown to preserve cargo integrity and bioactivity during room-temperature storage for up to 6 months [93]. Moreover, dissolving MNs can be engineered for sustained release tailored to complex pathological microenvironments. As exemplified by a dissolvable MN wound dressing with 800-μm needle tips designed to match dermal depth, this system enables precise intradermal delivery of platelet-derived exosomes, avoiding the physical barriers that limit topically applied counterparts while simultaneously functioning as a sustained-release depot that continuously releases anti-inflammatory and pro-angiogenic factors to promote long-term wound healing [94]. Despite these advancements, the utility of dissolving MNs for live-cell delivery is constrained by 2 intrinsic limitations. Encapsulated mammalian cells suffer substantial viability loss during fabrication due to osmotic and mechanical stresses inherent to solvent evaporation and polymer consolidation. Simultaneously, the incorporation of bulky cellular cargoes disrupts the polymeric network, precipitating a marked reduction in needle hardness and consequent mechanical failure upon skin insertion. Overcoming this trade-off between payload capacity and tissue penetrability thus demands fundamental innovations in both material design and fabrication methodologies [95,96].

### Porous MNs

With interconnected microvoids distributed across the needle matrix, porous MNs have become a promising tool for cellular therapeutics and localized regeneration [97]. These systems are typically fabricated from mechanically reinforced and biocompatible materials to compensate for the inherent fragility of their porous architecture, thereby ensuring adequate insertion capability and structural stability [98]. Various manufacturing strategies, including template replication, micromolding, and porogen-assisted techniques, have been developed to construct controllable porous geometries capable of accommodating cellular cargoes [97]. Relative to conventional solid MNs, porous variants offer distinct advantages for cell-associated delivery. Their interconnected internal cavities not only provide sufficient space for cargo accommodation but also facilitate nutrient diffusion, metabolite exchange, and sustained localized retention following administration [97]. These attributes are particularly advantageous for cell therapy, where preserving viability and post-delivery biological activity remains a major challenge [10]. Recent investigations have expanded the application of porous MNs toward direct cell encapsulation and localized regenerative therapy. In one example, bio-inspired porous MN arrays were engineered for stem cell-based diabetic wound treatment using template replication combined with particle etching. Poly(ethylene glycol) diacrylate (PEGDA) and gelatin methacryloyl (GelMA) were employed in constructing porous structures with interconnected pores of approximately 50 μm in diameter, dimensions sufficient to support ADSC encapsulation while sustaining proliferation, migration, and metabolic activity. Moreover, the microarchitecture geometry itself promoted localized cell retention and enhanced regenerative efficacy [99,100]. Collectively, these findings underscore the ability of porous MNs to function beyond passive delivery, serving as temporary niches that preserve cell viability and boost therapeutic outcomes.

### Hydrogel-forming MNs

HFMs, composed of crosslinked polymeric networks, have emerged as attractive platforms for cell-associated therapy owing to their unique ability to integrate mechanical delivery with localized biological regulation. Unlike conventional MNs that mainly function as passive transport devices, HFMs provide highly hydrated 3-dimensional (3D) microenvironments capable of preserving the integrity and bioactivity of sensitive cellular cargoes while enabling sustained and spatially controlled release [101]. After insertion, the hydrogel swells upon fluid absorption, producing dynamic diffusional routes that prolong tissue retention and increase the bioavailability of the therapeutic cargo. In addition, HFMs are commonly fabricated through micromolding strategies, which support reproducible manufacturing and scalable production for translational applications [102]. These characteristics make HFMs particularly suitable for cell-derived therapeutics that are vulnerable to mechanical damage and microenvironmental perturbation. Recent advances have extended HFM applications beyond direct cell transplantation toward the delivery of extracellular vesicles and secreted bioactive factors [103]. For example, a composite hydrogel MN system comprising methacrylated hyaluronic acid (HAMA) and decellularized adipose matrix (DAM) was developed for localized delivery of SC mitochondria-enriched microvesicles (Met-EVs) to accelerate chronic wound healing. The hydrated network protected vesicles from insertion-associated shear stress and enabled controlled release following matrix swelling [104]. Similarly, a GelMA-based hydrogel MN patch incorporating 3D-cultured MSC-derived exosomes was engineered for spinal cord repair, where the hydrogel matrix acted as a localized bioactive niche to preserve exosome activity and sustain therapeutic exposure at lesion sites [105]. These studies collectively demonstrate that HFMs can function not only as delivery carriers but also as temporary regenerative microenvironments that enhance the efficacy of cell-derived therapies. Nevertheless, their clinical translation remains challenged by limited mechanical robustness and the need to balance swelling behavior with efficient cargo transport.

### Hollow MNs

Hollow MNs, characterized by internal microchannels extending from the needle shaft to the tip opening, represent a unique class of MN systems capable of directly administering liquid formulations into target tissues. Unlike solid or dissolving MNs that rely on matrix-mediated release, hollow MNs enable active transport of therapeutic suspensions through integrated reservoirs, making them particularly attractive for cell-associated therapies that require high loading capacity and precise dosage control [106]. Compared with conventional needle injection, hollow MNs allow minimally invasive and localized administration while reducing tissue trauma, improving spatial distribution, and minimizing the mechanical stress and reflux commonly associated with direct injection of sensitive biological cargos [107]. These characteristics have recently expanded hollow MN applications beyond molecular therapeutics toward the delivery of cell-derived therapeutics and engineered biological systems. For example, Wang et al. developed a hollow MN platform for intratumoral delivery of tumor-derived EVs loaded with oncolytic viruses [108]. The system integrated a polymethyl methacrylate (PMMA) substrate with stainless-steel hollow MNs and functional membrane layers to establish a controllable delivery interface. Rather than directly introducing living therapeutic cells into tumors, the device employed virus-infected tumor cells as localized producers that continuously generated therapeutic extracellular vesicles within the reservoir. A size-selective membrane prevented cellular leakage while permitting sustained transport of nanoscale vesicles through the hollow channels. Combined with membrane-driven micropropulsion, the system achieved prolonged and spatially uniform delivery throughout dense tumor tissues, overcoming the elevated interstitial pressure and diffusion barriers that often limit conventional injection strategies [109]. Nevertheless, the clinical translation of hollow MNs remains limited by channel obstruction, mechanical instability, and inefficient transport of complex biological cargos. Future efforts should focus on optimizing device architecture and improving delivery precision and reliability for cell-associated therapies.

### Coated MNs

As a specialized solid MN subtype, coated MNs carry therapeutic payloads coated onto the needle tips, typically as suspensions or dispersions. Diverse fabrication methodologies have been employed to produce these systems, ranging from dip-coating and micromolding to advanced 3D printing techniques, where the uniformity and stability of the applied coating serve as critical quality attributes (CQAs) dictating dosing precision. Upon skin penetration, this configuration facilitates immediate interaction with ISF, promoting rapid dissolution of the coating and ensuring prompt drug absorption [110,111]. This rapid-release profile makes them highly advantageous for the transdermal administration of macromolecules like peptides and vaccines. However, current exploration for live-cell delivery is overwhelmingly restricted to dissolving or hydrogel-forming MNs, which inherently provide a 3D hydrated microenvironment compatible with cell viability [112]. In stark contrast, the formulation and fabrication of coated MNs rely on a desiccation process to solidify the payload on the matrix surface; this “drying” phase, compounded by fluid shear forces and a complete lack of nutrient media, inevitably triggers catastrophic cell death in fragile lineages such as SCs or T cells [111]. Although direct live-cell delivery via coated MNs remains largely unfeasible, this technology can effectively potentiate cell-based therapies through indirect immunomodulation [84]. For instance, a recent study utilized high-strength poly(l-lactic acid) (PLLA) MNs to assemble antigen–CpG immune-polyelectrolyte multilayers (iPEMs) directly onto the needle surfaces without carriers, enabling high-density loading. This system delivers the components to skin DCs, markedly elevating costimulatory signals and driving potent T cell activation and expansion in vivo [113]. Consequently, while the solid core and desiccated surface of coated MNs preclude the trafficking of intact, viable cells, they remain highly competitive as platforms for exosomes or cytokines intended to modulate endogenous cellular functions.

### Core–shell MNs

Core–shell MN designs represent highly sophisticated, dual-compartment architectures featuring a central internal matrix encapsulated by a peripheral shielding layer. Structurally, this spatial partitioning allows for a strategic division of labor where the core typically functions as a hospitable reservoir tailored to accommodate labile biological agents, whereas the rigid shell provides the requisite mechanical support and controls release kinetics [114,115]. In cell-based therapies, traditional single-component MNs suffer from a severe physical contradiction. Maximizing polymer concentration or crosslinking density to achieve the mechanical rigidity needed for skin penetration simultaneously suffocates cellular cargo, compressing mesh sizes and generating lethal osmotic or shear stresses that trigger cell apoptosis. Core–shell architectures elegantly bypass this historical trade-off by decoupling mechanical penetration requirements from the cellular survival microenvironment [116]. For instance, a core–shell MN configuration has recently been reported for the stable delivery of fibroblasts [117]. In this design, a dense poly(lactic-co-glycolic acid) (PLGA) outer layer serves as the load-bearing shell, preventing structural failure during tissue penetration. Conversely, the inner core comprises a highly hydrated, photo-crosslinked GelMA hydrogel that mimics the natural ECM. By isolating fragile fibroblasts within this soft, isotonic hydrogel core, researchers successfully shielded them from violent compressive and lateral shear forces during skin puncture. Furthermore, this aqueous microenvironment bypassed the destructive desiccation phases common in conventional dissolving MNs, maintaining over 80% cellular viability for up to 7 d. Thus, core–shell MN designs create a specialized niche that meets the needs of both adherent cells like MSCs and fibroblasts and fragile immunocytes such as CAR-T and NK cells via compartmentalization in a supportive hydrogel core shielded by a rigid shell [118]. This dual-phase design effectively resolves the longstanding trade-off between mechanical penetration capability and cellular viability, positioning core–shell MNs as precision-controlled platforms for transdermal cell delivery.

### Cryomicroneedles

CryoMNs represent a pioneering transdermal platform that leverages the phase-transition properties of a frozen aqueous matrix to deliver living cells without the chemical crosslinking, osmotic stress, or desiccation damage inherent to conventional polymeric systems. While frozen, the ice framework provides the rigidity required to breach the skin, and once inserted, it quickly liquefies, releasing viable cells into the dermal environment [119]. A critical consideration in cryoMN fabrication is the preservation of cell viability during freezing, which necessitates the inclusion of cryoprotectants. Dimethyl sulfoxide (DMSO), a widely used permeating cryoprotectant, functions by inhibiting intracellular ice crystallization and stabilizing cellular membranes during freezing. However, DMSO concentration must be carefully optimized because higher concentrations soften the ice matrix and compromise structural integrity during demolding, whereas lower concentrations fail to adequately protect cells from intracellular ice damage, resulting in precipitous viability loss [82,120]. To circumvent this concentration-dependent dilemma, recent studies have introduced sucrose as a nonpenetrating cryoprotectant that increases extracellular osmolarity, thereby drawing water out of cells and reducing intracellular ice formation while preserving membrane integrity. This dual-protectant strategy combines a low dose of 2.5% DMSO with sucrose in a phosphate-buffered saline base and effectively reconciles the conflicting requirements of mechanical robustness and cytoprotection. In fabrication, cells suspended in this optimized cocktail are cast into a polydimethylsiloxane mold and allowed to sediment into the needle tips via gravity, followed by a sequential gradient freezing regimen from −20 to −196 °C [82]. Hence, cryoMNs offer an ideal paradigm for delivering shear-sensitive mammalian cells such as MSCs and T cells, although their translational potential remains constrained by cold-chain dependency and a narrow clinical application window.

### Design principles of MNs for cell delivery

The 8 MN subtypes described above exhibit pronounced differences in their cell delivery suitability, which arise from variations in material composition, internal architecture, and mode of action. A comparative evaluation across multiple performance dimensions, encompassing cell loading capacity, post-delivery viability, protection mechanisms, release kinetics, and manufacturing scalability, elucidates distinct strengths and limitations that guide rational platform selection (Table 1).

**Table 1.** Comparative benchmarking of microneedle platforms for viable cell delivery

MN types	Representative materials	Cell loading capacity and location	Cell viability and living microenvironment	Advantages in cell delivery	Challenges in cell delivery
Solid	Stainless steel, titanium, and polymer	Cells are almost impossible to be directly encapsulated.	Most of the materials do not possess the microenvironment necessary for cell survival.	Pretreatment confers adequate mechanical rigidity for tissue penetration.	Lacks a protective polymeric matrix for cells and is largely obsolete for live cargos.
Dissolving	Water-soluble, biodegradable materials such as PVP, PVA, HA	Cells are directly encapsulated within the polymer matrix.	Fabrication method of solvent evaporation and polymer consolidation may cause substantial viability loss.	The poke-and-release approach permits controlled payload release without residual polymer deposition.	Restricted to fragile living cell cargo and slow polymer dissolution limits delivery kinetics.
Porous	Silicon, polymer, ceramic, metal (with interconnected pores)	Cells are largely hosted inside interconnected, microscale sponge-like internal void networks	Porous structures provide ample space for nutrient and metabolite exchange to support cell survival and ensure retention.	Interconnected macropores facilitate rapid nutrient and oxygen exchange and support efficient cell migration.	Reduced mechanical strength due to high porosity and hence limited for tissue penetration.
Hydrogel-forming	Swelling polymers based on crosslinked hydrogel networks such as GelMa and PEGDA	Cells are well encapsulated within a 3D hydrated polymeric network.	Hydrogel networks preserve the bioactivity and integrity of sensitive cellular cargoes.	Hydrated 3D network mimics native ECM, preserving sensitive cargo bioactivity and enabling sustained release.	Swelling behavior reduces needle stiffness, potentially hindering efficient skin insertion and controlled cargo release.
Hollow	Silicon and metallic materials including titanium and stainless steel	Cells are suspended in a liquid vehicle and loaded inside.	Cells experience no chemical or thermal stress during manufacturing and hence maintain viability.	Internal microchannels enable high-volume loading and minimized shear stress.	High risk of needle clogging by cell aggregates or tissue debris during insertion.
Coated	Core: Rigid metal and polymer, coating: soluble polymers like HA and PVP	Cells are localized solely to the exterior of the MN shafts.	The desiccation process with fluid shear forces inevitably triggers cell death.	Solid core guarantees excellent mechanical strength for penetration.	Extremely limited loading capacity due to thin surface area and the dehydration drying step risks cell death.
Core–shell	Shell: Rigid polymers like HA, core: hydrogels like GelMa	Cells are effectively embedded in the hydrogel-filled inner core.	Hydrogel core provides nutrition and suitable living environment, while shell preserves cells from possible damage.	Dual-compartment design decouples mechanical penetration, while soft hydrogel core preserves cells.	Complex multi-step fabrication and potential risk of structural delamination at the interface.
Cryo-	Freezing cell suspensions such as PBS and cryoprotectants like DMSO	Cells are uniformly embedded within the solid ice matrix.	Cells bypass dehydration and thermal stress during fabrication and preserve extremely high viability.	Cryopreserved ice matrix entirely bypasses chemical crosslinking and UV exposure, while cryoprotectants preserve cellular integrity.	Demands strict, uninterrupted cold-chain transport and rapid, specialized clinical handling.

#### Cell-specific challenges and MN design criteria

Advancing stem-, immune-, and adipose cell-based therapies toward clinical use requires a fundamental rethinking of delivery biomaterials. Unlike conventional small-molecule drugs, living cellular therapeutics possess large physical dimensions, exhibit exquisite sensitivity to mechanical shear forces, and impose stringent metabolic demands that must be met during and after administration [117]. To preserve cell viability following incorporation into MN platforms, natural biopolymers capable of recapitulating key characteristics of the ECM microenvironment are commonly selected. GelMA, HA, and carboxymethyl chitosan (CMCS) are frequently used as hydrogel matrices owing to their high water-retention capacity and inherent cell-adhesive motifs, including Arg–Gly–Asp (RGD) sequences and matrix metalloproteinase-cleavable sites [99,121,122]. Critically, cell encapsulation demands exceptionally mild and cytocompatible crosslinking conditions. Conventional MN fabrication parameters, such as elevated temperatures or exposure to aggressive organic solvents, must be strictly avoided. Current strategies therefore favor low-exposure photopolymerization or multi-network ionic crosslinking to circumvent cytotoxicity. CryoMNs therefore provide a distinctive strategy, in which cells are immobilized within a frozen lattice under cryogenic conditions, allowing cryoMNs to entirely circumvent chemical crosslinking and preserve near-complete cellular integrity upon in situ thawing [82].

Different therapeutic cell types impose distinct design requirements on MN platforms, necessitating a rational cell-MN matching strategy. Anchorage-dependent cells, such as MSCs and fibroblasts, require an ECM-like adhesive substrate, rendering hydrogel-forming, core–shell, or porous MNs most suitable [100,104]. Shear-sensitive immunocytes, including CAR-T and NK cells, demand gentle encapsulation environments to maintain viability, for which core–shell and cryogenic MNs are particularly appropriate [123]. Antigen-presenting cells like DCs benefit from co-delivery with adjuvants, favoring coated or porous architectures [113]. SC spheroids, with their 3D configuration, require large interconnected pores for accommodation, making porous MNs the optimal choice [100]. This cell-specific matching framework thus provides the foundation for rational MN platform selection in targeted therapeutic applications.

#### Fabrication methodologies and mechanical constraint optimization

The clinical translation of cell-loaded MNs presents a critical engineering challenge, as fabrication methodology fundamentally governs both needle structural integrity and post-production cellular viability. Traditionally, micromolding and solvent casting have served as mainstream approaches for dissolving, porous, and hydrogel-forming MNs due to their operational simplicity [124]. However, for live-cell therapeutics, conventional solvent casting can impose substantial biological stresses, including prolonged dehydration and cumulative compressive drying stresses, all of which may compromise cell membrane integrity and induce apoptosis. To overcome these limitations, additive manufacturing technologies, collectively known as 3D printing, have expanded design possibilities beyond traditional molding. Digital light processing (DLP) and stereolithography (SLA) belong to the category of vat-photopolymerization 3D printing methods and enable high-precision fabrication of complex MN geometries, yet their reliance on ultraviolet (UV) photoinitiators and the potential cytotoxicity of unreacted photo-monomers present challenges for direct cell encapsulation [107,125]. Fused deposition modeling (FDM), a material extrusion-based method, provides a low-cost option for thermoplastic polymers but requires high-temperature extrusion exceeding 180 °C, fundamentally precluding direct cell encapsulation. Two-photon polymerization (2PP), another 3D printing approach with ultrahigh resolution, achieves sub-micrometer precision for nano-topological features, although its low throughput and prohibitive costs limit clinical scalability. Extrusion-based bioprinting, which formulates shear-thinning bioinks, represents an alternative approach where rheological optimization can mitigate processing-induced shear stress [107].

Beyond fabrication-induced trauma, cell-loaded systems must withstand the mechanical constraints imposed during skin insertion. The incorporation of living cells introduces spatial discontinuities within polymer networks. These cell bodies may act as structural defects or stress concentrators, potentially compromising needle tip aspect ratio and compressive strength, which can lead to buckling upon encountering stratum corneum resistance. To resolve these competing demands, structural optimization strategies have been developed. Core–shell architectures effectively decouple mechanical penetration from cellular protection. As demonstrated in a recent study, a core–shell MN system comprising a fibroblast-laden GelMA hydrogel core surrounded by a PLGA shell maintained cell viability above 80% after 7 d in vitro [117]. CryoMNs offer an alternative strategy that entirely bypasses polymer-induced deformation. By forming a rigid ice-crystal lattice under controlled freezing, the frozen matrix provides sufficient mechanical stiffness to breach the skin barrier. Upon penetration, the ice melts rapidly in situ, enabling a release that preserves near-complete post-thaw cellular viability [82].

#### Translational feasibility and emerging frontiers

When benchmarked against alternative clinical modalities, MN platforms offer distinct multi-parametric advantages tailored for cell therapy. Compared to systemic intravenous administration, MNs enable localized and targeted deposition, circumventing poor tissue homing like off-target systemic toxicity [101]. Relative to direct intramyocardial or intratumoral injection, they provide a minimally invasive alternative that reduces procedural trauma and fluid bolus washout while improving spatial distribution. Unlike injectable hydrogels or macroscale scaffolds that require invasive surgical implantation, MN patches can be painlessly self-administered and removed with negligible tissue damage [126,127]. Nevertheless, MNs face limitations in loading capacity compared to bulk hydrogels, and their deployment is primarily confined to accessible transdermal or transmucosal sites. Catheter-based delivery allows access to deep organs but entails greater invasiveness, cost, and procedural complexity. The optimal performance of MNs thus depends on the therapeutic context, excelling in scenarios requiring localized, minimally invasive, and repeated administration of cell therapeutics to superficial or surgically accessible tissues [128].

Looking forward, several emerging frontiers are expected to broaden the clinical applicability of MN-assisted cell therapy. The integration of biosensing capabilities into MN platforms could enable real-time tracking of the activity of delivered cells and local tissue responses, enabling closed-loop therapeutic feedback [1]. Progress in smart materials enables disease-associated triggers including reactive oxygen species (ROS), matrix metalloproteinases, or pH gradients, thereby achieving spatiotemporally precise deployment synchronized with pathological progression [96,129]. Furthermore, the convergence of MN technology with artificial intelligence-driven manufacturing and patient-specific design, particularly through 3D printing, promises to accelerate the customization of MN geometries and material compositions for individualized treatment regimens [125]. Addressing these core translation-related barriers, including scalable production, regulatory harmonization, and durable storage stability, will be essential for converting encouraging preclinical results into clinically applicable therapies.

## MN-Mediated Cell Therapy for the Treatment of Organ Diseases

### MN-mediated cell therapy for skin diseases

Functioning as the most extensive physiological system in humans, the cutaneous layer acts as a formidable anatomical shield against external insults [130]. Skin diseases are highly prevalent and manifest with visible symptoms, including dermatitis, acne, wounds, and psoriasis [131]. Recent years have witnessed substantial progress in investigating cellular interventions as a functional alternative strategy for cutaneous pathologies beyond conventional medications [132]. Notably, the FDA has approved the transplantation of autologous fibroblasts for cosmetic purposes [133]. Cellular suspensions derived from the epidermis have demonstrated clinical efficacy in the rectification of dyspigmentation and the acceleration of closure in persistent ulcerations and traumatic injuries. Moreover, advances involving genetically modified cells, noncutaneous cells, and allogeneic cells have opened promising avenues for correcting skin disorders and repairing skin damage [134,135]. However, the broad clinical integration of cellular-based interventions is currently constrained by logistical hurdles, specifically the need to transport cells to targeted sites without compromising their viability or functionality [136]. To address these limitations, MN devices are being investigated as a delivery system capable of transporting therapeutic cells through microchannels created upon insertion [131,137].

#### MN-mediated cell therapy for skin tissue regeneration and wound healing

**Fig. 6.**
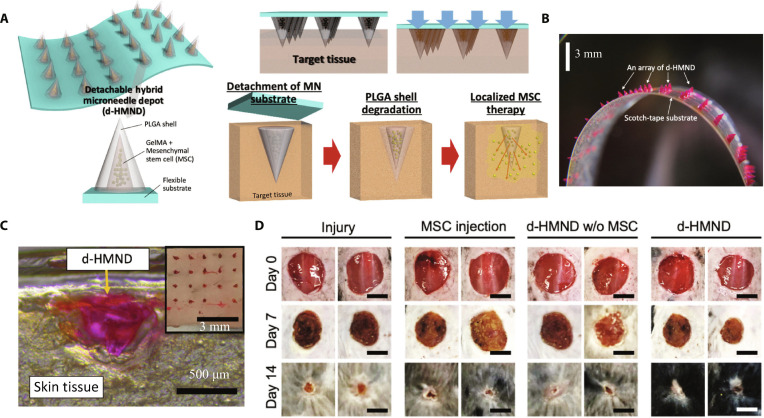
MN-mediated cell therapy for wound healing and skin tissue regeneration. (A) Schematic depicting the MSC-loaded d-HMND for injury recovery. (B) Visual representation of the d-HMND featuring a PLGA outer layer stained with red dye. (C) Micrograph of murine cutaneous tissue following d-HMND administration (utilizing rhodamine B for visualization). (D) Comparative photographic evidence comparing wound closure among different treatment cohorts (scale bars, 10 mm). Reproduced with permission from Ref. [138]. Copyright 2020 WILEY-VCH Verlag GmbH & Co. KGaA, Weinheim.

MSC-based therapies delivered via MNs have been widely researched due to their potent paracrine signaling and multi-lineage differentiation potential in tissue restoration. To address the insufficient retention and rapid viability loss of cells delivered through conventional routes, Lee et al. [138] fabricated a separable composite MN reservoir (d-HMND) designed to locally deliver viable, intact MSCs to wound sites. This platform features a biocompatible GelMA hydrogel core matrix that acts as a pro-survival niche, providing crucial mechanical shielding and nutritional support to sustain the encapsulated MSCs in their living state. Coupled with a detachable PLGA outer substrate, the system enables precise implantation into the deep dermis (Fig. 6A to C). Upon patch separation, the cell-laden GelMA micro-arrays remain embedded within the wound bed to mediate sustained therapeutic action. The d-HMND platform enabled dose-sparing cell delivery, sustained local cell viability, and expedited wound healing in a time-dependent manner (Fig. 6D) [138]. In a related study, Yuan and colleagues [139] engineered a GelMA/PEGDA MN loaded with human umbilical cord endothelial cell (HUVEC)-derived exosomes. The structural design of the dual-polymer matrix optimized exosomal cargo bioavailability, enabling effective epidermal barrier penetration and subsequent angiogenesis to accelerate wound repair.

**Fig. 7.**
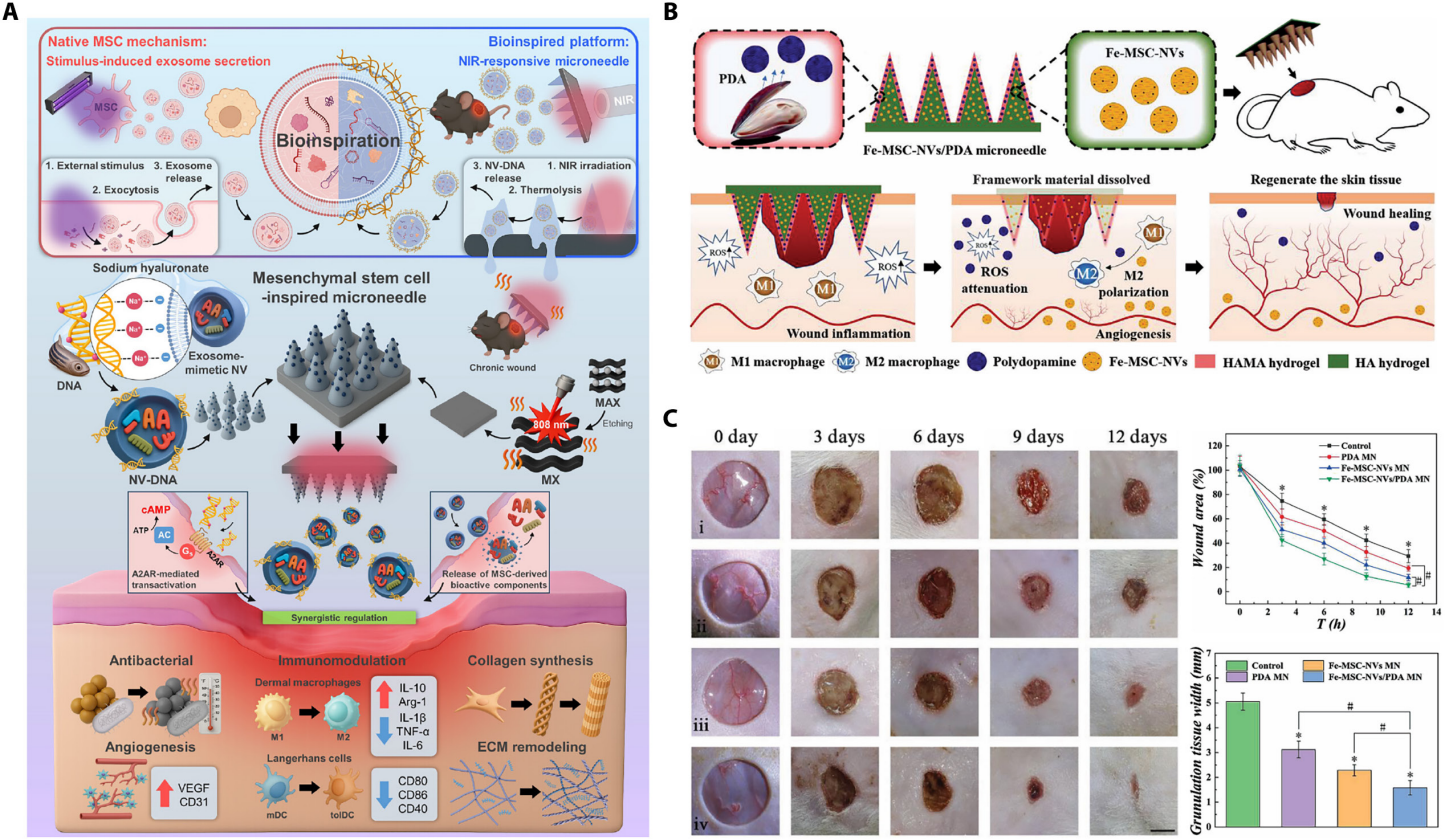
MN-mediated cell therapy for chronic diabetic wound healing. (A) The schematic illustrates an NIR-responsive dissolvable MN designed to mimic the triggered paracrine activity of MSCs functionalized with DNA sequences of salmon origin. This system enables controlled release to drive immune system regulation, neovascularization, and stromal reconstruction, ultimately accelerating the repair of cutaneous tissue. Reproduced with permission from Ref. [96]. Copyright 2025 The Authors, published by Wiley-VCH GmbH. (B) Diagrammatic representations show the core–shell MN co-loaded with PDA and Fe-MSC-NVs for chronic wound management, including the mode of regenerative action. (C) Photographic evidence compares the healing outcomes in the control and various MN-treated groups at sequential time points (scale bars, 5 mm). The accompanying data track the reduction in wound surface area over a 12-d period and provide histomorphometric data regarding the breadth of the granulation tissue on the final day of evaluation. Reproduced with permission from Ref. [140]. Copyright 2022 The Authors, published by Wiley-VCH GmbH.

Beyond basic wound management, multi-compartmentalized MN platforms have proven exceptionally versatile in orchestrating synergistic, stage-specific interventions in complex pathological settings, notably chronic diabetic wounds. Chronic wounds are trapped in a self-perpetuating pathological state marked by sustained inflammation, excessive oxidative stress, and deficient vascularization. To break this impasse, Moon et al. [96] introduced an advanced biomimetic, dissolvable MN platform that encapsulates MSC-derived nanovesicles (MSCNVs) functionalized with DNA sequences of salmon origin, coupled with a near-infrared (NIR)-responsive photothermal release mechanism. Upon NIR stimulation, the system triggered spatiotemporal release of paracrine-mimicking nanovesicles. This effectively induced M1 to M2 macrophage polarization and up-regulated VEGF, thereby promoting fibroblast migration, proliferation, and neovascularization (Fig. 7A). Adopting a comparable core–shell HA micro-array design, Ma et al. [140] leveraged a sequential-release structural architecture to optimize the delivery of iron-loaded MSC nanovesicles alongside antioxidant polydopamine (PDA) nanoparticles (Fig. 7B). This multi-functional orchestration bypassed the pathological barriers of diabetic ulcers, importantly accelerating wound closure and granulation tissue organization via the coordinated mitigation of oxidative stress and stimulation of neovascularization (Fig. 7C).

#### MN-mediated cell therapy for superficial skin cancer

**Fig. 8.**
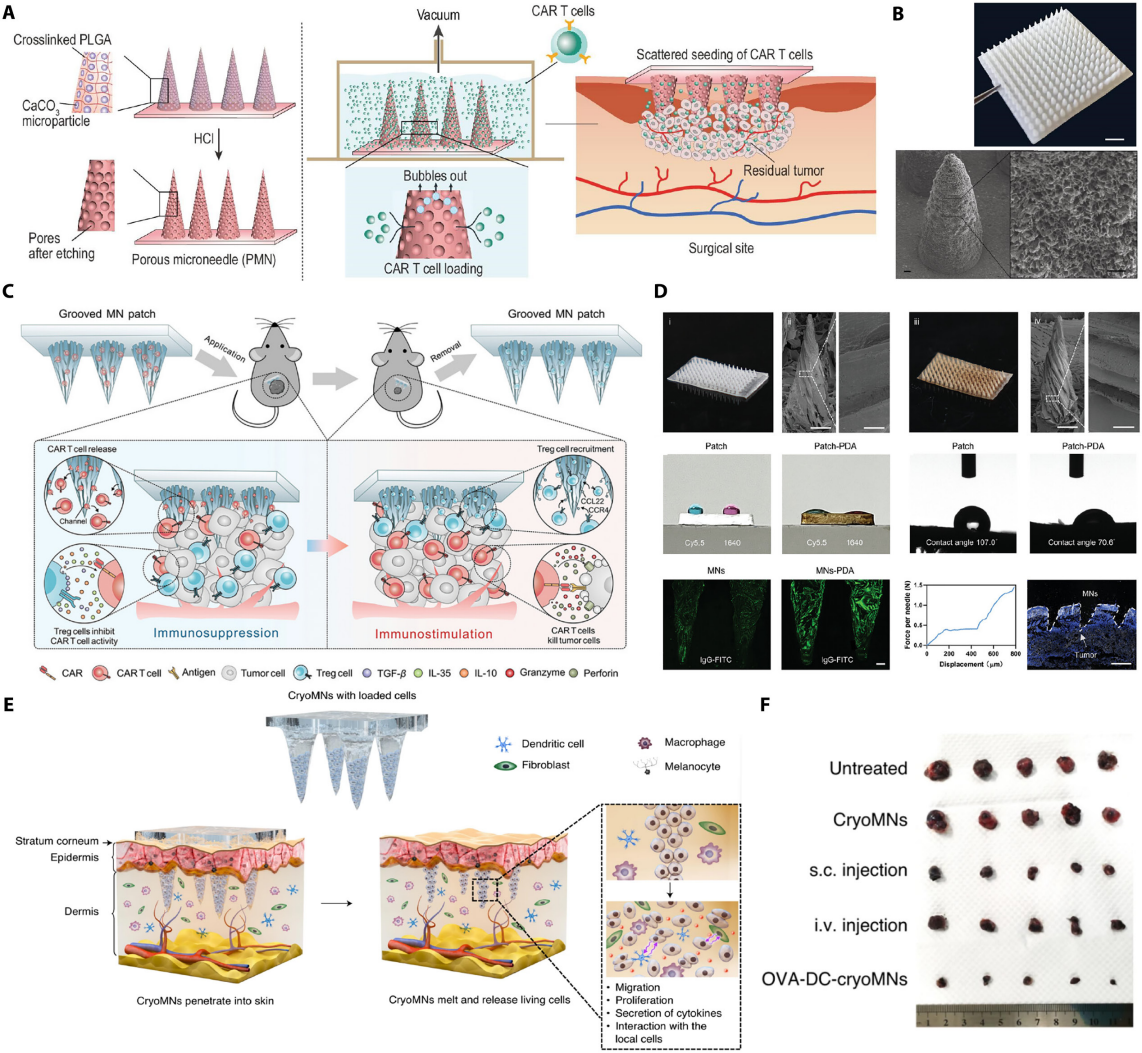
(A) Conceptual diagrams outlining the sequential fabrication of porous MNs integrated with CAR-T cellular payloads. The engineered devices were subsequently implanted directly into the neoplastic matrix following the surgical procedure. (B) The illustrative macroscopic image of the porous MNs (scale bar, 2 mm) and corresponding scanning electron microscopy (SEM) micrographs illustrating the internal matrix architecture (scale bars, 50 μm). Reproduced with permission from Ref. [142]. Copyright The Authors 2021. Published by Oxford University Press on behalf of China Science Publishing & Media Ltd. (C) Schematic of the MN patch-mediated intratumoral T cell delivery and T_reg_ diversion. (D) Characterization of the grooved MNs for enhanced T cell adoptive immunotherapy. Reproduced with permission from Ref. [88]. Copyright 2024 Wiley-VCH GmbH. (E) Diagrammatic representations of the transdermal cell delivery utilizing cryoMNs. (F) Images of excised tumors harvested from 5 mice per experimental cohort on day 26. Reproduced with permission from Ref. [82]. Copyright The Authors, under exclusive license to Springer Nature Limited 2021.

Therapeutic strategies based on adoptive cell transfer (ACT), including DC vaccines and CAR-T cells, represent a paradigm shift in oncology. However, their clinical efficacy against solid cutaneous tumors, such as melanoma, is severely crippled by compact ECMs that restrict immune-cell penetration, together with a strongly immunosuppressive TME dominated by T_reg_ cells. Traditional systemic administrations often culminate in poor tumor homing and systemic toxicities [141]. To shatter these physical and immunological barriers, researchers have transitioned to biomaterial-assisted local delivery. Addressing the prerequisite of high-dose infiltration, Li et al. [142] engineered a highly porous MN micro-array utilizing a CaCO_3_ composite and PLGA. This architecture endowed the MNs with robust mechanical integrity for cutaneous penetration, while the interconnected porous networks enabled an unprecedented loading capacity of ∼22,000 CAR-T cells per needle (Fig. 8A and B). In post-surgical melanoma models, this design allowed encapsulated cells to rapidly egress and infiltrate surrounding tissues, markedly suppressing tumor regrowth compared to traditional intratumoral injections.

Beyond providing porous escape routes, the structural topography of the MN can be innovatively manipulated to actively remodel the TME. Addressing this, Zhou et al. [88] developed a chemokine CCL22-functionalized grooved MN patch to simultaneously bypass physical and immunosuppressive barriers. Engineered via an ice-templated method, the microscale grooved channels on the needle surface dramatically expanded the T cell loading capacity. Mechanistically, the MN core serves as a localized reservoir enabling intralesional administration of tumor-specific T cells, whereas the surface-grafted CCL22 establishes a localized cytokine concentration gradient. This gradient actively traps and diverts endogenous immunosuppressive T_reg_ cells out of the tumor core and onto the MN surface, thereby reducing the intratumoral T_reg_ ratio and augmenting the therapeutic efficacy of ACT (Fig. 8C and D). Furthermore, to safeguard delicate cells against manufacturing stress, Chang et al. [82] employed a stepwise cryogenic micro-molding technique to develop cryoMNs for delivering ovalbumin-pulsed DCs (OVA-DCs) (Fig. 8E). By maintaining a protective, ice-templated microenvironment, the cryoMNs preserved cellular viability and antigen-presenting functionality, triggering robust systemic antitumor immunity that substantially outperformed standard systemic routes (Fig. 8F). Expanding the deployment of such structurally and compositionally optimized MNs to transport living, engineered cell lineages into other epithelial malignancies represent a highly promising frontier for future oncological research.

#### MN-mediated cell therapy for inflammatory skin diseases

**Fig. 9.**
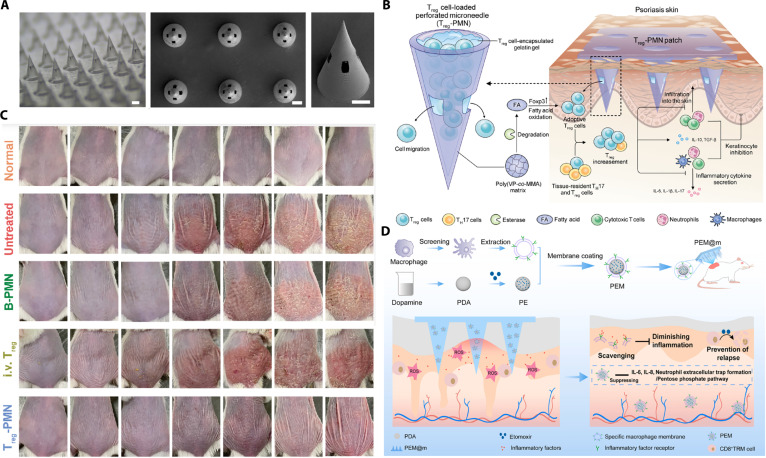
(A) Conceptual diagrams detailing the porous micro-array-facilitated adoptive transfer of T_reg_ cells for psoriatic lesions. (B) Illustrative SEM micrographs of the perforated MN structures are provided, with magnified views showing scale bars of 300 μm and 200 μm, respectively. (C) Representative images depicting the dorsal skins from psoriasis-bearing mice in each treatment group on days 0 to 6 (from left to right). B-PMN, blank MNs; i.v. T_reg_, systematic injection of T_reg_ cells; T_reg_-PMN, treatment with T_reg_ cell-loaded MNs. Reproduced with permission from Ref. [146]. Copyright 2023 The Authors. (D) Illustrative model depicting the MN-mediated delivery of macrophage membrane-modified nanoparticles for psoriasis therapy. Reproduced with permission from Ref. [148]. Copyright 2025 Elsevier Ltd.

Essential for preserving cutaneous immune balance, T_reg_ cells are central to systemic stability. Consequently, disruption of T_reg_ function is a prominent factor in the development of chronic autoimmune disorders, with psoriasis as a notable example [143]. However, inadequate site-specific homing and insufficient regional sequestration of adoptive T_reg_ cells have severely stymied the clinical integration of cellular protocols for circumscribed cutaneopathies [144]. Traditional systemic infusions or local injections fail to achieve sustained accumulation within inflamed lesions, leading to rapid cellular clearance or off-target immunosuppression. To shatter these bottlenecks, recent structural innovations have leveraged multi-compartmentalized MNs to provide a protective, pro-survival niche for intact immune cells [145]. Addressing this, Zhang et al. [146] developed an innovative perforated core–shell MN patch, featuring a polymeric envelope synthesized from methyl methacrylate (MMA) and vinyl propionate (VP). The optimized poly(VP-co-MMA) ratio conferred robust mechanical strength to the MNs to ensure seamless epidermal penetration, while an interconnected gelatin matrix encapsulated within the shell successfully preserved T_reg_ cell viability (Fig. 9A and B). Uniquely, the gradual degradation of the polymeric shell releases lipid-based metabolites directly into the inflamed microenvironment, a biochemical mechanism that further potentiates the immunosuppressive function of the delivered cells. In murine psoriasis models, this intralesional delivery substantially attenuated epidermal hyperplasia and inflammatory cell infiltration compared to traditional injections (Fig. 9C).

Furthermore, the intricate inflammatory landscape of psoriasis is driven by a complex network of multi-pathway mediators, making therapies targeting single cytokines highly susceptible to disease relapse [147]. To address this, current research has pivoted toward biomimetic cell-derived therapies as an acellular, multi-targeted alternative. Huang et al. [148] proposed a biomimetic MN patch utilizing macrophage membranes as cellular “decoys” to neutralize multiple inflammatory cascades simultaneously. By coating core PDA nanoparticles with plasma membranes derived from activated macrophages, the system leverages naturally expressed surface receptors to capture and sequester a broad spectrum of excessive pro-inflammatory factors. This MN-based platform importantly enhanced local retention and tissue penetration while minimizing systemic exposure, achieving superior therapeutic and preventive efficacy compared to the clinical topical agent calcipotriol (Fig. 9D).

Although MN-facilitated delivery of therapeutic cells has demonstrated considerable potential for wound healing and skin cancer treatment, persistent barriers still hinder clinical translation. First, the therapeutic efficacy of transplanted cells remains highly dependent on the local pathological microenvironment. Microenvironmental cues, including oxidative stress, inflammation, and pH imbalance, may impair cell survival or alter cellular phenotypes after transplantation, yet methods for real-time monitoring of these dynamic changes remain limited. Second, increasing cell loading often compromises the mechanical strength of porous MNs, reducing their insertion efficiency, particularly in hyperkeratotic or fibrotic skin. Future development should therefore focus on multifunctional MN platforms capable of integrating precise delivery with dynamic microenvironmental regulation. The incorporation of flexible biosensors and microfluidic technologies may enable real-time monitoring of local physiological signals, allowing responsive and controlled release of therapeutic cells according to disease progression. In addition, advances in biomaterial engineering and intelligent MN design may facilitate more effective regulation of local immune responses, ultimately supporting the advancement of individualized and precision cellular therapies for cutaneous diseases.

### MN-mediated cell therapy for endometrial repair

**Fig. 10.**
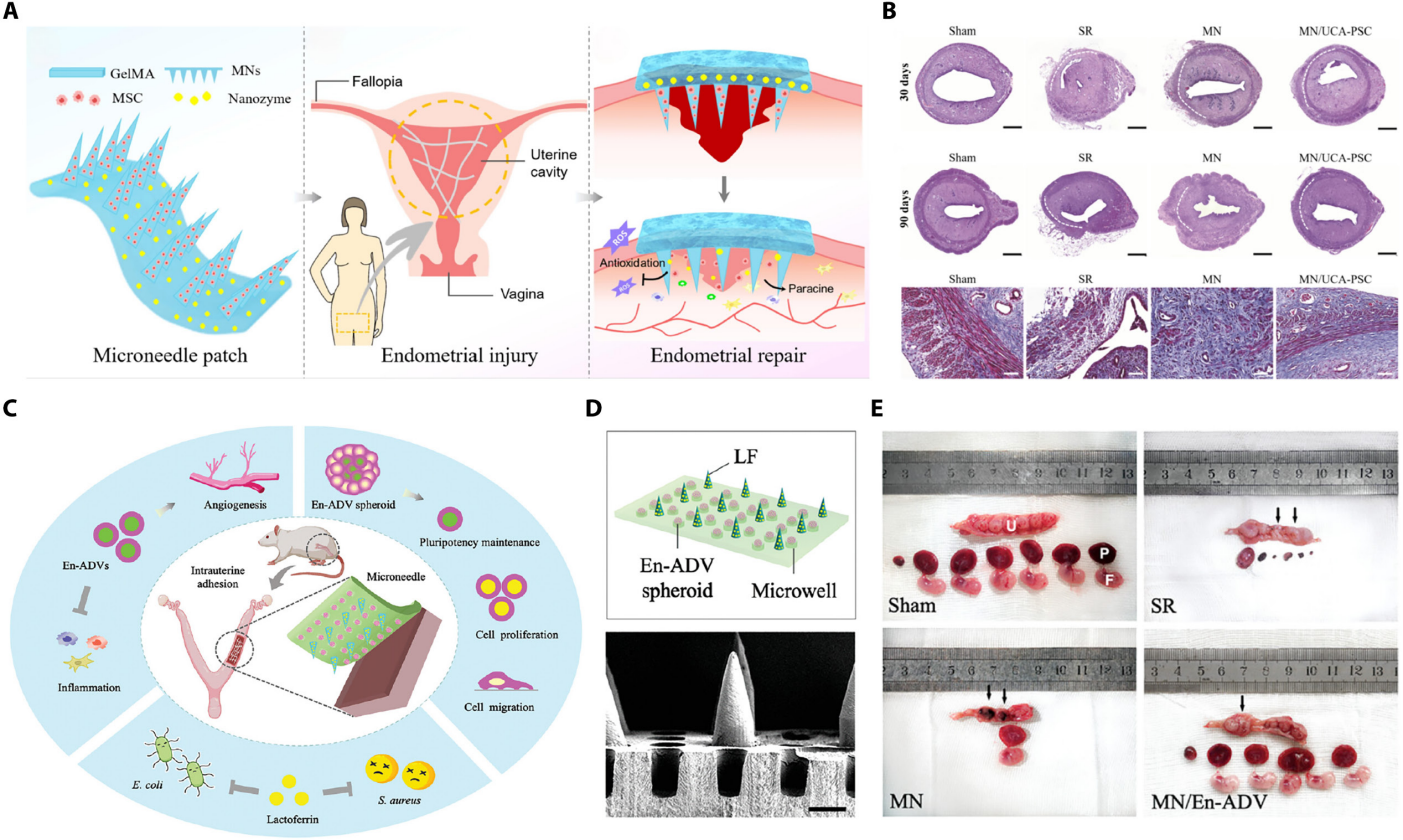
(A) Schematic illustrations of SC-loaded antioxidant nanozyme MNs for the application of in situ endometrial repair. (B) Histological morphology of the regenerative uterine horns and their collagen deposition in different groups (SR, spontaneous regeneration group; scale bars corresponding to 500, 500, and 50 μm, distributed across the first to the last row). Reproduced with permission from Ref. [151]. Copyright 2022 Elsevier B.V. (C) Overview of MN-mediated En-ADV administration for intrauterine tissue restoration. (D) Schematic and characterization of MNs loaded with En-ADV spheroids (scale bar, 200 μm). (E) Illustrative captures characterizing the physiological restoration of the reconstructed uterine tissue at a 90-d post-surgical interval. Reproduced with permission from Ref. [152]. Copyright 2022 Wiley-VCH GmbH.

Endometrial injury, characterized by disruption of the endometrial basal layer, is strongly linked to recurrent miscarriage and impaired fertility [149]. Although SC-based therapies have shown promise for endometrial repair, their clinical implementation is still constrained by inefficient site-specific localization, poor post-transplantation retention, and reduced cellular survival within the oxidative lesion microenvironment [150]. These limitations highlight that successful endometrial repair requires not only cell delivery but also sustained cellular activity and microenvironmental support. To address these barriers, Zhu and colleagues [151] engineered a GelMA-based micro-array for site-specific administration of umbilical artery-derived perivascular SCs (Fig. 10A). To further improve post-transplantation survival, cerium oxide (CeO_2_) nanozymes were incorporated into the platform to scavenge ROS, thereby alleviating oxidative stress and enhancing regenerative outcomes (Fig. 10B). In an independent study, Li and coworkers [152] engineered a porous GelMA-based MN designed to deliver human endometrial adventitial cells (En-ADVs) enabling in situ formation of 3D cellular spheroids within the porous architecture. This design enhanced cell proliferation, migration, and functional persistence, resulting in uterine regeneration and fertility restoration in a rat model of endometrial injury (Fig. 10C to E).

Although MN-mediated delivery addresses the high cell loss and poor engraftment of traditional intrauterine injections, further development faces unique biomechanical and translational scrutiny. First, MNs encounter anatomical dynamic instability because the uterus is a dynamic organ with cyclic contractions, endometrial shedding, and continuous mucus flushing. Current studies rely on static animal models and lack quantitative evaluations of MN resistance to slippage under intense uterine wall movement. Furthermore, long-term observations are missing regarding whether forced physical anchoring induces secondary mechanical injury or chronic sterile inflammation. Second, the diffusion depth of cells is limited in highly fibrotic tissue. The short needle length cannot fully penetrate severely adhered or fibrotic endometrium, leaving cells trapped in superficial layers and unable to activate deep adventitial cells for endogenous regeneration. Future development should prioritize biomimetic MN platforms that combine biomechanical compliance with spatiotemporally controlled therapeutic delivery. Incorporating wet-tissue adhesive and shape-memory properties may enable MN patches to achieve stable contact with the curved uterine surface while adapting to continuous uterine movement, thereby enhancing localized cell retention and treatment efficacy.

### MN-mediated cell therapy for heart repair

**Fig. 11.**
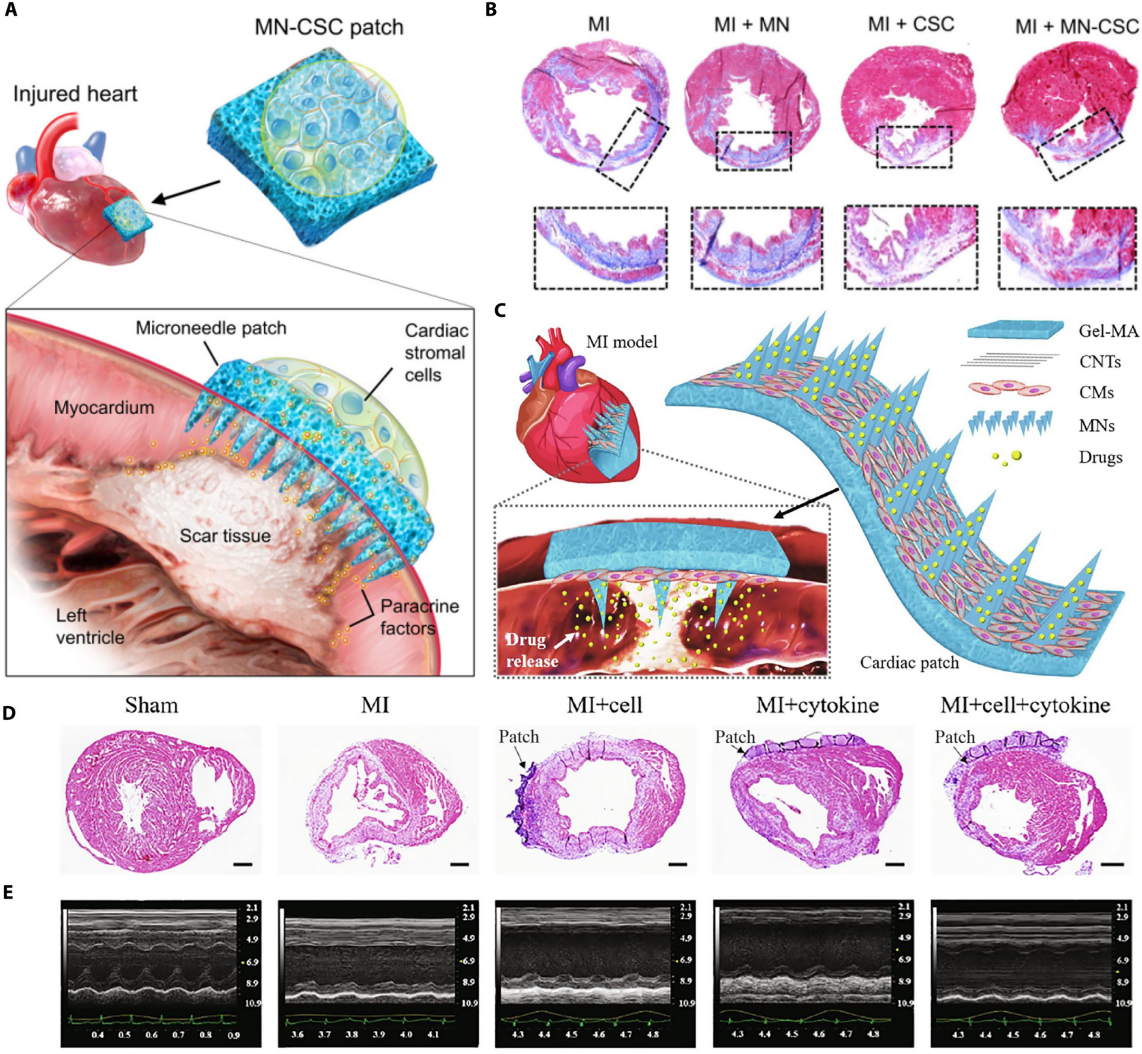
(A) Schematic illustrations of MN-mediated CSC therapy for heart repair. (B) Representative Masson images depicting the function of cardiac repair by MN-CSC 3 weeks after myocardial infarction. Reproduced with permission from Ref. [128]. Copyright 2018 The Authors. (C) Conceptual diagrams of an MN system incorporating both CNTs and CMs for infarcted heart therapy. (D) Representative hematoxylin and eosin (H&E) staining and (E) M-mode echocardiographic images from different groups following the therapeutic intervention (scale bars, 500 μm). Reproduced with permission from Ref. [156]. Copyright 2021 Elsevier B.V.

Cardiac injury constitutes an important contributor to global disease burden and mortality, with progression to heart failure frequently occurring as a consequence of the constrained regenerative capacity inherent to adult myocardial tissue [153]. Although cell-based therapies have demonstrated superior regenerative potential compared with conventional pharmacological interventions, their clinical efficacy is strongly constrained by inefficient delivery and poor post-transplantation persistence [154]. Conventional approaches, including intramyocardial injection and systemic administration, often suffer from low cell retention, heterogeneous spatial distribution, and procedural invasiveness, resulting in insufficient functional integration within injured myocardium [155]. Unlike regenerative applications in relatively static tissues, successful cardiac repair imposes additional requirements on delivery systems, including adaptation to continuous mechanical contraction, maintenance of cell viability, and establishment of synchronized communication with host cardiac tissue. To address these challenges, recent MN platforms have increasingly evolved beyond passive cell transport toward creating localized regenerative interfaces capable of supporting myocardial reconstruction. Tang et al. [128] reported a polymeric MN array in which cardiac SCs (CSCs) were incorporated within fibrin-containing PVA MNs (Fig. 11A). By establishing direct contact with injured myocardium, the platform improved local cellular persistence and facilitated sustained paracrine signaling, thereby promoting tissue repair and functional recovery (Fig. 11B). This strategy highlights the importance of maintaining prolonged cell–tissue interaction rather than relying solely on transient cell engraftment. Building upon this concept, Sun et al. [156] introduced a conductive MN platform integrating iPSC-derived cardiomyocytes (CMs) with an electroactive architecture (Fig. 11C). The platform consisted of a drug-loaded MN base, an intermediate layer containing conductive carbon nanotubes (CNTs), and a GelMA scaffold to guide cellular organization and enhance intercellular electrical communication. By promoting spatial alignment and synchronized contraction of transplanted CMs with native myocardium, this platform achieved improved physiological integration and superior cardiac repair outcomes (Fig. 11D and E).

Epicardial MN patches offer great potential for treating myocardial infarction, yet the harsh cardiac environment raises critical challenges. The primary concern is the unknown electrophysiological safety. Integrating conductive materials like CNTs promotes synchronous contraction, but their long-term retention may alter local electrical anisotropy and create arrhythmogenic foci. Current short-term animal studies lack clear safety boundaries. Additionally, MNs face cell capacity limitations. Infarction causes the loss of millions of cells, meaning the limited payload of MNs requires rational dosimetric evaluations for large transmural injuries. Future perspectives move toward wireless electro-active hydrogels and in situ cellular reprogramming. Future MNs will replace nondegradable fillers with biodegradable, myocardial-compliant hydrogels driven by wireless electromagnetic fields to promote mature sarcomere development. To overcome capacity limits, the strategy will shift from cell transplantation to in situ reprogramming, delivering specific transcription factors to convert scar fibroblasts directly into functional CMs, shifting the paradigm from physical replacement to biological remodeling.

### MN-mediated cell therapy for oral diseases

**Fig. 12.**
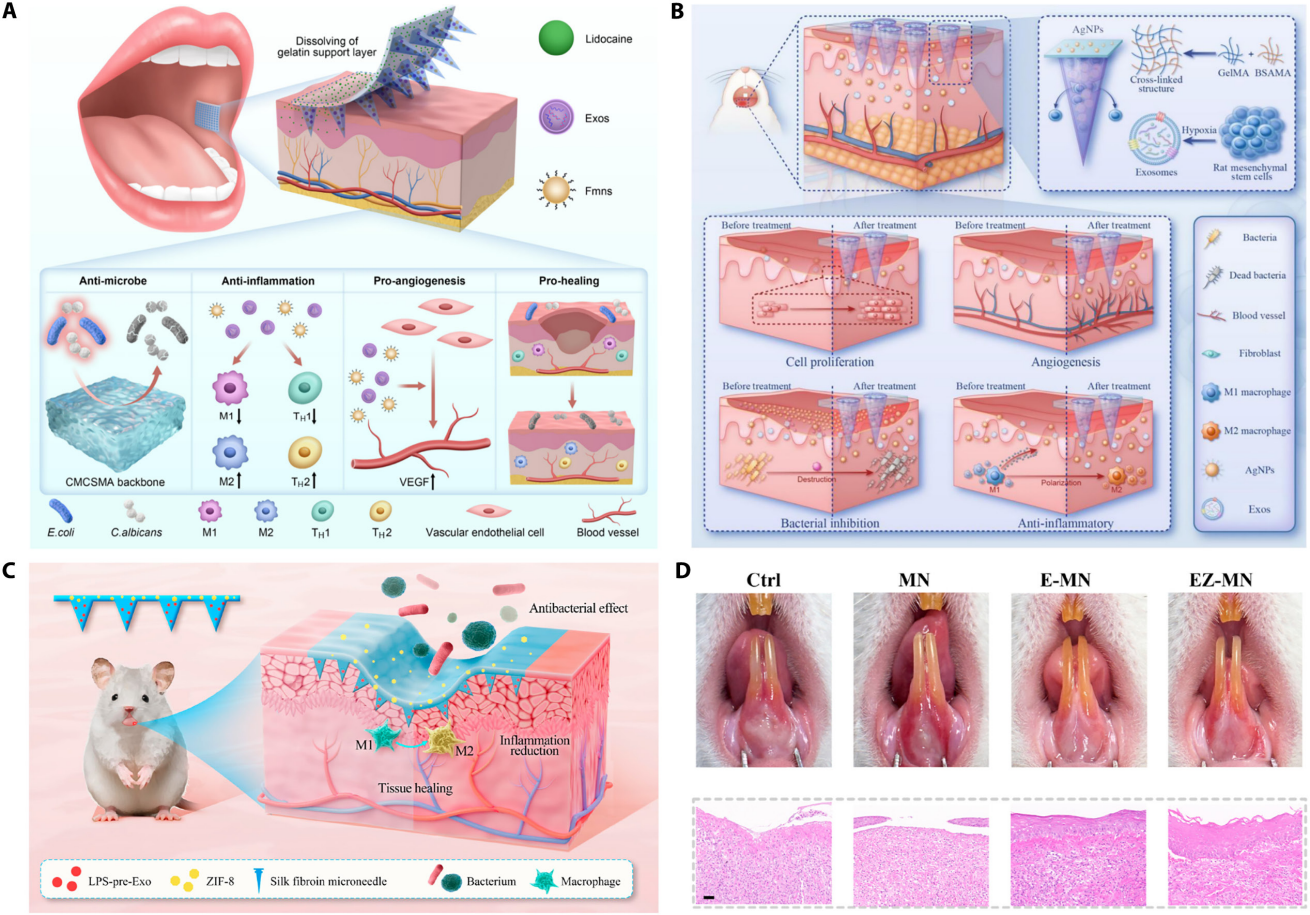
(A) Illustrative diagrams detailing the transmucosal delivery and administration of the multifunctional MNs. Reproduced with permission from Ref. [160]. Copyright 2024 Elsevier Ltd. (B) Graphical representations outlining antibacterial BSAMA/GelMA MNs loaded with AgNPs and exosome for facilitating oral ulcer healing. Reproduced with permission from Ref. [161]. Copyright 2024 Elsevier B.V. (C) Conceptual diagrams of an MSC-derived exosome-loaded MN system for oral mucosal lesion therapy. (D) Representative images and H&E staining of rat oral ulcer healing (scale bar, 50 μm). Reproduced with permission from Ref. [162]. Copyright 2024 American Chemical Society.

Oral mucosal lesions, particularly recurrent oral ulcers, present severe clinical challenges due to persistent inflammation, intense pain, and secondary microbial infections that disrupt chewing and articulation [157,158]. More recently, MSC exosomes have been recognized as a promising acellular therapeutic approach, offering superior immunomodulatory and regenerative capabilities compared to alternative vesicle sources [159]. However, the complex oral environment involves constant salivary flushing, mechanical shear, and enzymatic degradation, which severely compromise the retention and efficacy of conventional topical formulations [87]. To breach these physiological barriers, localized MN patches have been engineered to establish resilient bio-interfaces directly on the wet mucosal barrier. As an example, Chen and colleagues [160] developed a multifunctional MN platform using methacrylate carboxymethyl chitosan (CMCSMA) matrix to co-deliver exosomes derived from bone marrow MSCs (BMSCs) together with folate-conjugated magnetic nanoparticles (Fig. 12A). This polymeric architecture leveraged the mucoadhesive and inherent antibacterial properties of chitosan to enhance patch stability, enabling the sustained intramucosal diffusion of exosomes to accelerate epithelialization and angiogenesis. Crucially, the patch integrated a sacrificial gelatin layer for immediate local anesthetic release, providing rapid pain relief while mitigating secondary opportunistic infections. Similarly, Zeng et al. [161] fabricated a photo-crosslinked composite MN system blending GelMA and bovine serum albumin methacryloyl (BSAMA) to deliver silver nanoparticles (AgNPs) alongside exosomes derived from hypoxia-preconditioned MSCs (Fig. 12B). This synergistic system substantially accelerated wound healing by down-regulating pro-inflammatory cytokines and shifting macrophage polarization from M1 toward the reparative M2 phenotype. To further enhance cellular activity, Ge and colleagues [162] constructed a silk fibroin MN patch incorporating exosomes derived from lipopolysaccharide (LPS)-pretreated BMSCs (Fig. 12C). This inflammatory priming strategy importantly enhanced the anti-inflammatory potency and bacterial-clearing efficiency of the SC-derived vesicular cargo, leading to rapid ulcer healing and excellent histocompatibility (Fig. 12D).

Integrating MNs into the complex oral environment expands options for mucosal delivery and periodontal regeneration, but clinical practicality faces challenges. First, rapid mucosal renewal and continuous saliva flow cause a mismatch between the delivery window and tissue barriers. Hydrogel MNs often dissolve or displace due to tongue movement before fully releasing cells or exosomes, creating dosage uncertainty. Second, piercing the mucosal barrier risks carrying oral pathogens into deep tissues. Ensuring MNs do not trigger infections or micro-abscesses in immunocompromised patients remains an overlooked safety concern. Additionally, rapid cell inactivation in the warm, enzyme-rich oral cavity presents a major hurdle. Future perspectives will focus on ultra-fast anchoring and establishing safety barriers. Developing smart MNs that lock within seconds is essential to resist saliva washing and chewing forces. Upon insertion, the backing will dissolve instantly, leaving only the therapeutic needle tips anchored in the mucosa. Externally, the needle shell will integrate antimicrobial agents to create a sterile pathway during insertion, while the inner core delivers repair cells. Finally, 3D-printed custom MN scaffolds will enable precise, targeted delivery of different cells to revolutionize alveolar bone regeneration.

## Current Challenges and Future Perspectives

Notwithstanding encouraging preclinical outcomes, translating MN-mediated cell therapy into clinical practice remains constrained by challenges extending beyond therapeutic efficacy and device optimization. As living cell-loaded MN systems integrate active biological products with implantable biomaterials, successful implementation requires coordinated advances in manufacturing, storage, regulation, and clinical evaluation. Scalable and standardized manufacturing represents a major translational bottleneck. Current fabrication approaches, including manual micromolding and centrifugal loading, remain difficult to scale while maintaining reproducibility and cellular activity. To comply with current GMP requirements and regulatory expectations for cell-based products, manufacturing workflows must ensure batch-to-batch consistency while preserving cellular identity, potency, sterility, and structural integrity. Emerging technologies such as automated microfluidic dispensing and high-throughput bioprinting offer opportunities to improve scalability; however, these approaches must minimize prolonged ex vivo manipulation and mechanical stress that may impair cell function. In parallel, standardized CQAs, including cellular viability, loading uniformity, insertion performance, mechanical integrity, and release reproducibility, should be established to support quality control and reliable product release.

Storage and sterilization introduce additional complexity. Unlike conventional pharmaceuticals, living cell-loaded MN platforms are incompatible with standard terminal sterilization methods because these procedures can compromise cellular integrity and biological activity. Consequently, manufacturing is expected to rely on aseptic processing and closed production systems. Long-term preservation remains another unresolved challenge, as hydrogel-based matrices are susceptible to dehydration-induced loss of viability. Although cryopreservation and cryoMN strategies have demonstrated potential for maintaining cell activity, dependence on cold-chain transport and concerns regarding post-thaw functionality continue to limit clinical feasibility. Regulatory evaluation and clinical implementation further influence translational success. Living cell-loaded MN systems are likely to be regulated as combination products, requiring simultaneous assessment of both biological and device-related performance. Beyond biocompatibility and mechanical evaluation, regulatory approval may require comprehensive characterization of cellular potency, degradation behavior, sterility assurance, and long-term safety. Economic feasibility and trial design must also be considered, as conventional pharmacokinetic endpoints are insufficient for localized cell therapies. Future clinical studies should incorporate standardized assessment of retention, spatial distribution, therapeutic persistence, patient compliance, and long-term outcomes. Through integration of scalable manufacturing, regulatory standardization, and clinically relevant evaluation frameworks, MN-mediated cell therapy may ultimately progress toward clinically accessible regenerative medicine.

In summary, MN-mediated cell therapy offers a compelling strategy to address shortcomings of conventional delivery methods by enabling localized, minimally invasive administration of therapeutic cells and cell-derived products. As highlighted throughout this review, the diverse pathological characteristics of different diseases impose distinct requirements on MN design, including material composition, structural architecture, and release behavior. Increasing evidence indicates that therapeutic outcomes depend not only on efficient cellular delivery but also on the ability of MN systems to actively regulate local microenvironments through coordinated modulation of inflammation, tissue repair, vascularization, and functional integration. Despite substantial advances in preclinical studies, movement toward the clinic is still hindered by barriers involving scalable production, quality assurance, storage robustness, regulatory assessment, and clinical verification. Future development should therefore focus on programmable and multifunctional MN platforms that integrate precise cellular delivery with dynamic microenvironmental regulation. Continued progress in biomaterials, biofabrication, and translational science may ultimately enable MN-mediated cell therapy to mature into a clinically accessible strategy for regenerative medicine and precision therapeutics.
